# Multi-locus phylogeny of the catfish genus *Ictalurus* Rafinesque, 1820 (Actinopterygii, Siluriformes) and its systematic and evolutionary implications

**DOI:** 10.1186/s12862-023-02134-w

**Published:** 2023-06-28

**Authors:** Rodolfo Pérez-Rodríguez, Omar Domínguez-Domínguez, Carlos Pedraza-Lara, Rogelio Rosas-Valdez, Gerardo Pérez-Ponce de León, Ana Berenice García-Andrade, Ignacio Doadrio

**Affiliations:** 1 Laboratorio de Biología Acuática, Facultad de Biología, Universidad Michoacana de San Nicolás de Hidalgo, Ciudad Universitaria, Morelia, 58000 Michoacán México; 2 Forensic Science, Medicine School, National Autonomous University of Mexico, Circuito de la investigación científica s/n, Ciudad Universitaria, Coyoacan, 04510 CdMx Mexico; 3 Laboratorio de Colecciones Biológicas y Sistemática Molecular, Unidad Académica de Ciencias Biológicas, Universidad Autónoma de Zacatecas, Av. Preparatoria S/N, Campus Universitario II, Col. Agronómica, Zacatecas, C. P. 98066 México; 4 Instituto de Biología, UNAM, Circuito exterior s/n, Ciudad Universitaria, Coyoacán, C.P. 04510 D.F México; 5 Escuela Nacional de Estudios Superiores Unidad Mérida, Universidad Nacional Autónoma de México, Km 4.5 Carretera Mérida-Tetiz, Ucú, Yucatán México; 6 Laboratorio de Macroecología Evolutiva, Red de Biología Evolutiva, Instituto de Ecología, A.C. Carretera antigua a Coatepec 351, El Haya, Xalapa, 91070 Veracruz México; 7 Departamento de Biodiversidad y Biología Evolutiva, Museo Nacional de Ciencias Naturales, CSIC, c/José Gutiérrez Abascal 2, Madrid, E-28006 España

**Keywords:** Evolution, Freshwater fishes, Ictaluridae, North America, Taxonomic classification

## Abstract

**Background:**

*Ictalurus* is one of the most representative groups of North American freshwater fishes. Although this group has a well-studied fossil record and has been the subject of several morphological and molecular phylogenetic studies, incomplete taxonomic sampling and insufficient taxonomic studies have produced a rather complex classification, along with intricate patterns of evolutionary history in the genus that are considered unresolved and remain under debate.

**Results:**

Based on four loci and the most comprehensive taxonomic sampling analyzed to date, including currently recognized species, previously synonymized species, undescribed taxa, and poorly studied populations, this study produced a resolved phylogenetic framework that provided plausible species delimitation and an evolutionary time framework for the genus *Ictalurus*.

**Conclusions:**

Our phylogenetic hypothesis revealed that *Ictalurus* comprises at least 13 evolutionary units, partially corroborating the current classification and identifying populations that emerge as putative undescribed taxa. The divergence times of the species indicate that the diversification of *Ictalurus* dates to the early Oligocene, confirming its status as one of the oldest genera within the family Ictaluridae.

**Supplementary Information:**

The online version contains supplementary material available at 10.1186/s12862-023-02134-w.

## Background

Members of the order Siluriformes, commonly known as catfishes, have been the focus of evolutionary studies given their ancient origin during the early Cretaceous [1], but also as a result of their wide distribution and species diversity. Catfishes have inhabited salt, brackish, and freshwater areas on all continents [2, 3] and are considered a model system for the research of historical continental relationships and biogeographic patterns [3–7]. The evolutionary history of this group has been explored using different information sources [1, 8–10]. Their morphological particularities allow an extensive fossil record that provides evidence of their evolutionary and biogeographic history [11–13].

Within Siluriformes, the family Ictaluridae is considered to have evolved since the Eocene (ca. 65 million years ago: Mya) and is the only extant catfish group to occur in North America [1]. Ictaluridae currently comprises approximately 51 extant species belonging to seven genera: *Ameiurus* Rafinesque 1820, *Noturus* Rafinesque 1818, *Pylodictis* Rafinesque 1818, *Satan* Hubbs and Bailey 1947, *Trogloglanis* Eigenmann 1919, *Prietella* Carranza 1954, and *Ictalurus* Rafinesque 1820 [14]. Sixteen additional fossil species (one of the most complete fossil records of North American freshwater fishes) have also been reported [11]. Although molecular phylogenetic analyses have been conducted for species included in some ictalurid genera; i.e., *Noturus*, *Ameiurus*, and *Prietella* (11, 15–16), those of *Ictalurus*, the widest distributed genus in North America (from Canada to Belize; 11), have been poorly studied to date. The phylogenetic relationships and species composition within *Ictalurus* remain uncertain, leading to unstable classification. Several species have been synonymized, and individuals of some populations are even considered undescribed taxa [2, 17].

Lundberg [2] provided the most comprehensive morphological phylogenetic analysis of the genus *Ictalurus* in a study that resolved two clades: the *furcatus* group, which includes *Ictalurus furcatus* Valenciennes, 1840 and *Ictalurus balsanus* Jordan and Snyder, 1899, and the *punctatus* group, which includes *Ictalurus australis* Meek, 1904, *Ictalurus dugesii* Bean, 1880, *Ictalurus lupus* Girard, 1858, *Ictalurus mexicanus* Meek, 1904, *Ictalurus pricei* Rutter, 1896, and *Ictalurus punctatus* Rafinesque, 1818. However, the phylogenetic relationships within the *punctatus* group were unresolved. Although focused on studying higher-rank relationships, the most recent phylogenetic hypothesis, which included species of *Ictalurus* based on genetic sequences and morphological data, actually revealed *Ictalurus balsanus* as the earliest diverged species, followed by the divergence between *I. furcatus* + *I. meridionalis* and the *punctatus* group [13].

Although based solely on morphological evidence, some studies aimed at analyzing the taxonomic cohesiveness of certain widely distributed species suggest the existence of species complexes. Such is the case of *Ictalurus lupus* (2, 18–19), which occurs along the extensive Bravo River basin and other independent drainages along the Gulf of Mexico slope, such as the Cuatro Cienegas valley and Soto la Marina River. Further examples include *I. pricei*, with a distribution range comprising several river drainages along the Pacific slope of the Sierra Madre Occidental [20], and *I. dugesii*, for which two differentiated western populations have been proposed [17]. On the other hand, there is some controversy regarding species synonymies; Miller et al., [17] consider two synonymized species: *Ictalurus ochoterenai* de Buen, 1946 with *I*. *dugesii* and *Ictalurus meridionalis* with *I*. *furcatus*, while Gilbert [21] considers *Ictalurus meeki* Meek, 1902, a synonym of *I. pricei*; and Lundberg [2] considers *I. australis* a synonym of *I*. *punctatus*, whereas Fricke et al. [22] consider *I. australis, I. ochoterenai*, and *I. meridionalis* as valid species.

Based on the above, and the most complete taxonomic survey of described and putatively undescribed forms of *Ictalurus*, the present study infers molecular phylogenetic relationships among *Ictalurus* species, exploring their biogeographic evolutionary history in North American river drainages and resolving certain genus-related taxonomic uncertainties.

## Results

### Gene trees

We generated 192 novel sequences of *cytb*, 135 of *coxI*, 136 of *atpase8/6*, and 116 of *RAG1* (with no indels), for 97 localities across the distribution range of the *Ictalurus* species in North America (Table S1). In addition, 63 sequences of *cytb*, 71 of *coxI*, 2 of *atpase8/6*, and 3 of *RAG1* (including some species of *Ictalurus* as well as outgroups) were retrieved from GenBank (Table S1). The final amplicon lengths for *cytb, coxI, atpase8/6*, and *RAG1* were 1092, 609, 861, and 1035 bp, respectively. Assessment of substitution saturation based on the entropy-based index indicates that when the index of substitutional saturation (**Iss**) is smaller than the critical indices of symmetrical (**Iss.cSym**) and asymmetrical (**Iss.cAsym**) substitutional saturation, the gene sequences have experienced little substitution saturation, implying that these sequences are useful (23–24). The results of the saturation test in the present study show that the values of **Iss** were significantly lower than those of **Iss.cSym** and **Iss.cAsym** for the three positions at four loci (*P* < 0.05; Table S2). This indicates that there was little saturation and that the four data sets correspond to useful sequences.

**Fig. 1:**
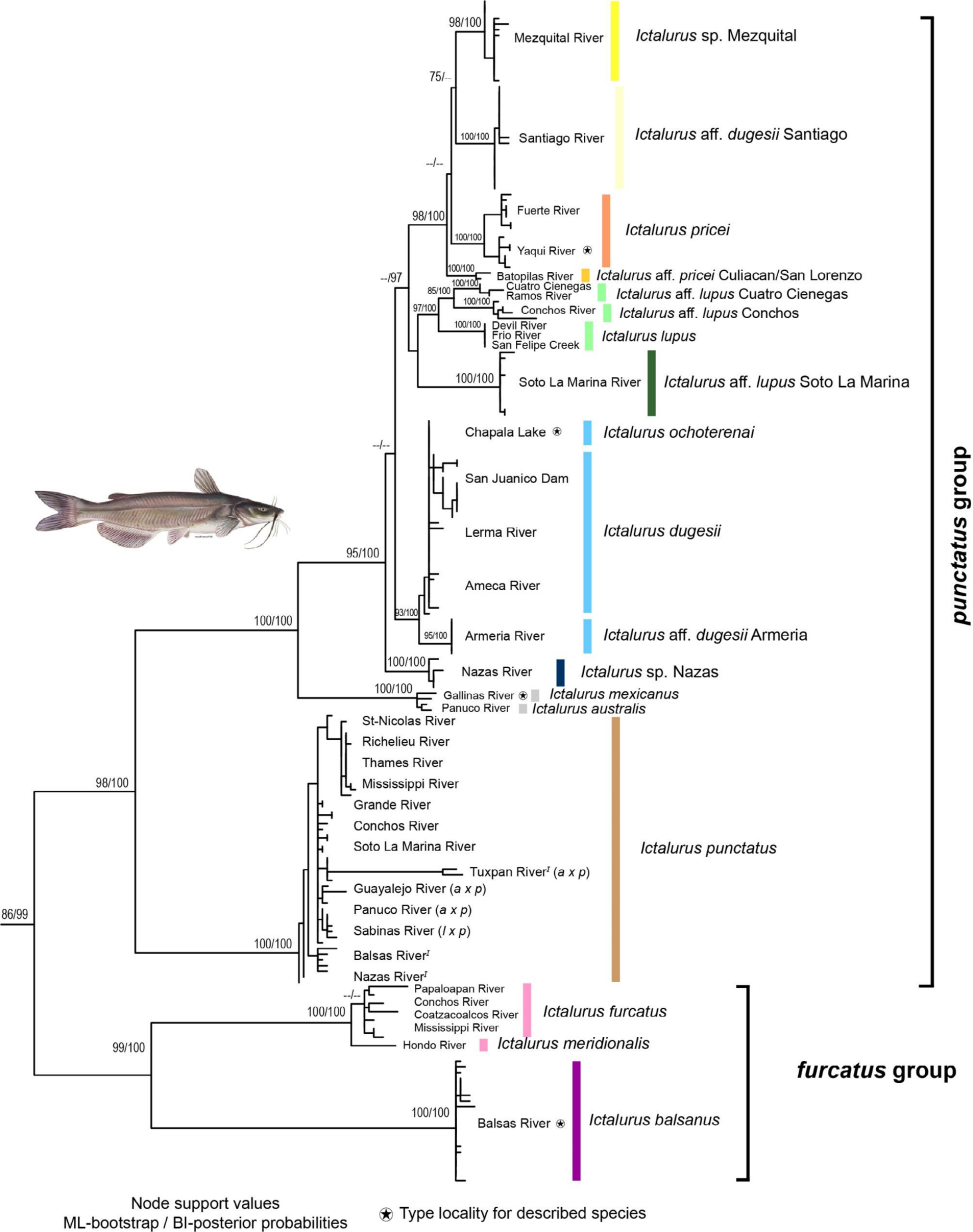
Maximum likelihood hypothesis of the genus *Ictalurus* based on the mitochondrial gene *cytb*. The mt/nuc discordance in *I. australis* x *I. punctatus* (*a x p*) and *I. lupus* x *I. punctatus* (*l x p*), as well as non-native populations (^*I*^), are indicated in tip labels. Tip names are taxa included in our study and tip colors match the geographic range of each valid species shown in Fig. 4. Channel catfish illustration from Duane Raver at U.S. Fish & Wildlife Service [https://www.fws.gov/]. Outgroups are not shown

Using maximum likelihood (ML) and Bayesian inference (BI), the phylogenetic relationships with the genes *cytb* (Fig. 1), *coxI* (Fig. S1), and *RAG1* (Fig. S2) revealed two well-supported clades, corresponding to the *furcatus* and *punctatus* groups. For *cytb*, the *furcatus* group recovered two clades corresponding to the recognized species *I. balsanus* and *I. furcatus* whereas, with *coxI*, we recovered three clades corresponding to *I. balsanus*, *I. furcatus*, and *I. meridionalis*. As with *cytb*, for *RAG1*, *I. meridionalis* was nested within *I. furcatus* (Fig. S2).

For *coxI* and *cytb*, the *punctatus* group produced two main clades, one formed by the populations of *I. punctatus* and the other grouping the rest of the species/populations. In this latter clade, five supported groups were recovered: (i) populations assigned to *I. mexicanus* and *I. australis*, (ii) populations of the Nazas basin, (iii) populations of *I. lupus, I.* aff. *lupus* from the Soto la Marina River, *I.* aff. *lupus* from Cuatro Cienegas and *I.* aff. *lupus* from the Conchos River, (iv) populations assigned to *I. dugesii, I. dugesii* from Armeria, and *I. ochoterenai*, and (v) populations assigned to *I. pricei, I.* aff. *dugesii* from the Santiago River, *Ictalurus* sp. from Mezquital, and *I.* aff. *pricei* from the Culiacan/San Lorenzo Rivers. Within this clade of the *punctatus* group, the clade *I. mexicanus/I. australis* was the earliest diverged lineage (Fig. 1; Fig. S1). *RAG1* showed no resolution in the relationships among species, indicating *I. punctatus* as the early-diverged species, which is related to a clade formed by at least three recovered subclades in a polytomy. The mitochondrial *atpase8/6* gene did not yield the same interrelationships. Firstly, the *furcatus* group was not recovered as a monophyletic group, since *I. balsanus* emerged as the early diverged clade, related to a clade formed by the remaining species of *Ictalurus* (Fig. S3); and secondly, low resolution was found within the *punctatus* group (Fig. S3).

*Ictalurus* aff. *dugesii* Armeria was resolved as a monophyletic group with *cytb* and *coxI*, but only with the first locus showing reciprocal monophyly in relation to *I. dugesii* samples (Fig. 1; Fig. S1). The remaining populations of *I. dugesii* are not supported as reciprocal monophyly based on the three mitochondrial loci (Fig. 1, S1, and S3). *Ictalurus ochoterenai* (a species previously considered as a synonym of *I. dugesii*) was not recovered as a monophyletic group, since it was also found to be nested within the *I. dugesii* clade using all genes (Fig. 1; Fig. S1-S3). The differences between *cytb* and *coxI* consisted of the topology based on the first locus: the populations of *I.* aff. *lupus* from the Conchos River, *I.* aff. *lupus* from Cuatro Cienegas valley, and *I.* aff. *lupus* from the Soto la Marina River were recovered as reciprocally monophyletic groups in relation to *I. lupus*, a clade considered as the *lupus* complex in the present study (Fig. 1). The *coxI* phylogeny showed the first two populations to be part of *I. lupus* and related to *I.* aff. *lupus* from the Soto la Marina River (Fig. S1). Regarding the relationships based on the *cytb*, a clade was formed by the differentiated populations of *I.* aff. *dugesii* from the Santiago River, *Ictalurus* sp. from the Mezquital River, *I.* aff. *pricei* from the Culiacan/San Lorenzo Rivers, and the recognized species *I. pricei*. In this study, we refer to this clade as the *pricei* complex, which showed strong clade support values and was closely related to the *lupus* complex (Fig. 1). Relationships based on *coxI* showed the *pricei* complex as monophyletic, but the relationships among the monophyletic clades included therein were unresolved (Fig. S1). Another difference from the topology obtained with *cytb* is that the *pricei* complex was related to *I. dugesii* in the *cox1* tree (Fig. S1).

With the gene *RAG1* (Fig. S2), the following phylogenetic mitochondrial/nuclear (mt/nuc) discordance is highlighted: *(1)* the two specimens from the Guayalejo River within the Panuco basin, morphologically identified as *I. australis* but assigned as *I. punctatus* with mitochondrial loci (Fig. 1; S1; and S3), were nested within *I. mexicanus* samples (Table 1; Fig. S2); *(2)* two small morphologically undetermined specimens, one from the Santa Maria River in the Panuco basin, and the other from the Pantepec River in the Tuxpan basin, were assigned to *I. punctatus* using mitochondrial loci (Fig. 1; S1, and S3) but were nested within *I. mexicanus*/*australis*, (Table 1; Fig. S2); and *(3)* another undetermined specimen from the Sabinas River in the Bravo River basin, confirmed as *I. punctatus* using mitochondrial loci (Fig. 1; S1, and S3), was nested within the *lupus* complex (Table 1; Fig. S2).

**Table 1:** Individuals that presents mito-nuclear discordance and morphological incongruence

Morphological species	Mitochondrial loci	Nuclear locus (*RAG1*)	Locality	Basin
*I. australis*	*I. punctatus*	*I. australis*	Guayalejo River	Panuco
*I. punctatus*	*I. punctatus*	*I. australis*	Santa Maria River	Panuco
*I. punctatus*	*I. punctatus*	*I. mexicanus*	Pantepec River	Tuxpan
*I. punctatus*	*I. punctatus*	*I. lupus*	Sabinas River	Bravo

**Fig. 2:**
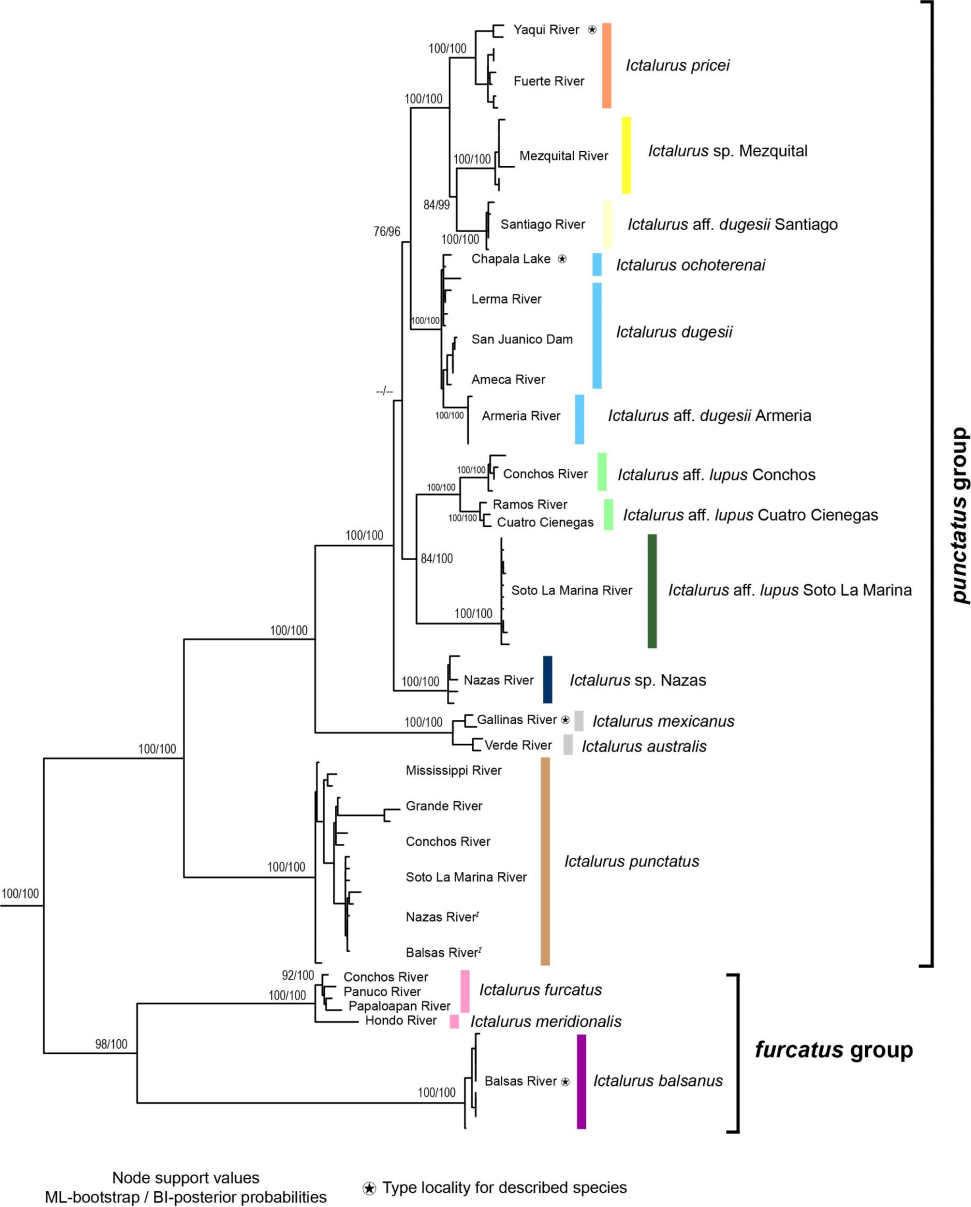
Maximum likelihood hypothesis of the genus *Ictalurus* based on the Concatenated matrix. Non-native populations (^*I*^) are indicated in the tip labels

In the concatenated analysis, the specimens *I. lupus* and *I.* aff. *pricei* from the Culiacan/San Lorenzo Rivers were excluded, including the individuals caused by the above phylogenetic discordance. Thus, the concatenated analysis included the recognized species and six previously differentiated populations (*I.* aff. *dugesii* Santiago, *Ictalurus* sp. Mezquital, *Ictalurus* sp. Nazas, *I.* aff. *lupus* Conchos, *I.* aff. *lupus* Cuatro Cienegas, and *I.* aff. *lupus* Soto la Marina) and their relationships were resolved with high nodal support (Fig. 2). Overall, the concatenated analysis clearly showed the early diversification of the species in the *furcatus* and *punctatus* groups. The phylogenetic tree also evidenced the recovery of two species complexes (those of *pricei* and *lupus*) and supported the position of *Ictalurus dugesii* as the sister taxon of the *pricei* complex, as suggested by analysis of the independent genes *coxI*, and *RAG1* (Fig. S1 and S2).

Uncorrected *p* genetic divergences ranged from 2.2 to 11.6% for *cytb*, 2.6 to 10.9% for *coxI*, 2.2 to 12.7% for *atpase8/6*, and 0.2 to 1.8% for *RAG1* (Tables S2-S5). In particular, the comparisons between different monophyletic groups presented herein exceeded the genetic divergence range within the species level limit observed for ictalurids using *cytb* (1.8–3.6%) [16, 25–27]. Genetic divergences between previously synonymized species and their respective valid nominal species (i.e., *Ictalurus ochoterenai* – *I. dugesii*, *I. australis* – *I. mexicanus* and *I. meridionalis* – *I. furcatus*) were very low, reaching 0.2, 0.5, and 1.5% for *cytb*; 0.1, 0.3, and 1.3% for *coxI*; 0.1, 1.8, and 0.5% for *atpase8/6*; and 0.2, 0.5, and 0.2% for *RAG1*, respectively. The genetic divergences of known differentiated populations with respect to closely related species included the following: divergences between *I. dugesii* and *I.* aff. *dugesii* Armeria were 0.9, 0.4, 0.6, and 0.2%, for *cytb*, *coxI*, *atpase8/6*, and *RAG1*, respectively. Divergences between *I. dugesii* and *I.* aff. *dugesii* Santiago were 2.1, 2.6, 1.9, and 0.2%, for *cytb*, *coxI*, *atpase8/6*, and *RAG1*, respectively. The genetic divergences among the four monophyletic groups recovered within the *pricei* complex ranged from 1.6 to 1.9% for *cytb*, 1.6 to 2% for *coxI*, 1.4 to 2.1% for *atpase8/6*, and 0.1 to 0.3% for *RAG1* (Tables S2-S5), whereas the divergence among the four monophyletic groups recovered within the *lupus* complex ranged from 1.9 to 3.4% for *cytb*, 1.1 to 1.8% for *coxI*, 1.1 to 4% for *atpase8/6*, and 0.1 to 0.3% for *RAG1* (Tables S2-S5).

### Species trees

**Fig. 3:**
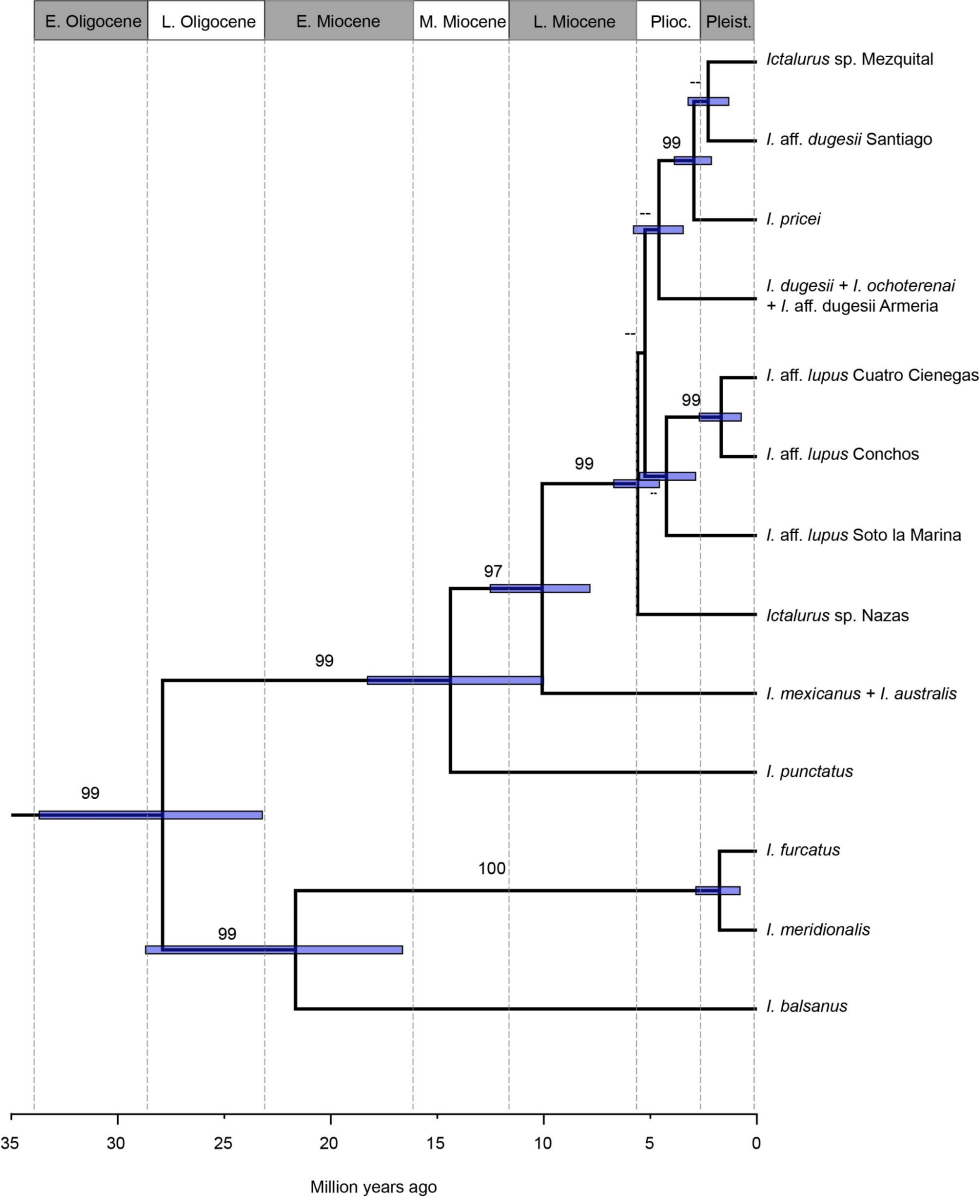
Species tree with divergence times of the genus *Ictalurus*. Values on the branch correspond to posterior probabilities; only values ≥ 95% are shown. Blue node bars correspond to the 95% highest posterior density

Nucleotide substitution rates derived from the time-calibrated tree were 3.054 × 10^− 3^ for *cytb*, 2.695 × 10^− 3^ for *coxI*, 2.877 × 10^− 3^ for *atpase8/6*, and 2.897 × 10^− 4^ for *RAG1* substitution/site/per million years. Although presenting low nodal support, the species tree based on the four loci (Fig. 3) showed the same topology as that yielded by the concatenated analysis (Fig. 2). Likewise, for the time-calibrated tree (Fig. S4), the species tree recorded similar divergence times (Fig. 3). The most recent common ancestor (MRCA) of *Ictalurus* was dated at ca. 27.9 Mya (23.2–33.9 Mya, 95% high posterior density), during the Oligocene. Most of the diversification events within *Ictalurus* occurred during the Late Miocene-Early Pliocene (Fig. 3).

### Bayesian species delimitation

Two Bayesian Phylogenetics and Phylogeography (BPP) analyses were conducted; A10 (comparison of species delimitation models induced on a given “guide” tree [27]) and A11 (joint comparison of species delimitation/assignment and tree inference of unguided tree [27]), both of which yielded similar results. In the A10 analyses, almost all the splits involving the recognized species (*Ictalurus pricei*, *I. dugesii*, *I. mexicanus*, and *I. punctatus*) and differentiated populations (*I.* aff. *pricei* Culiacan/San Lorenzo, *Ictalurus* sp. Nazas, and *I.* aff. *lupus* Soto la Marina) were supported by the four scenarios (Table 2). For the splits involving *I. balsanus*, *I. lupus*, and the lineages pair *I.* aff. *dugesii* Santiago *– Ictalurus* sp. Mezquital, only the second (IGθ = 3, 0.002, IG τ_0_ = 3, 0.004) and fourth (IGθ = 3, 0.002, IG τ_0_ = 3, 0.4) scenarios were supported when ancestral population size was small (Table 2). Finally, the splits of *I. furcatus – I. meridionalis*, and the differentiated population pair *I*. aff. *lupus* Cuatro Cienegas *– I*. aff. *lupus* Conchos, were unsupported (Table 2).

**Table 2:** Posterior probabilities from A10 analysis showing the support in the splits involving the species/lineages

**Priors**	***pricei*** **complex**			
	*Ictalurus* sp. Mezquital / *I.* cf. *dugesii* Santiago		*I. pricei* / remaining clades	*I.* cf. *pricei* Culiacan/San Lorenzo / remaining clades
θ(3, 0.2) τ(3, 0.4)	0.06*		**1.00**	**1.00**
θ(3, 0.002) τ(3, 0.004)	**1.00**		**1.00**	**1.00**
θ(3, 0.2) τ(3, 0.004)	0.13*		**1.00**	**1.00**
θ(3, 0.002) τ(3, 0.4)	**1.00**		**1.00**	**1.00**
	**differentiated lineages of** ***lupus*** **complex**			
	*I.* cf. *lupus* Cuatro Cienegas / *I.* cf. *lupus* Conchos		*I. lupus /* remaining clades	*I.* cf. *lupus* Soto la Marina */* remaining clades
θ(3, 0.2) τ(3, 0.4)	0.01*		0.13*	**1.00**
θ(3, 0.002) τ(3, 0.004)	0.50*		**1.00**	**1.00**
θ(3, 0.2) τ(3, 0.004)	0.06*		0.30*	**1.00**
θ(3, 0.002) τ(3, 0.4)	0.16*		**1.00**	**1.00**
	***furcatus*** **group**			
	*I. furcatus / I. meridionalis*		*I. balsanus* / remaining clades	
θ(3, 0.2) τ(3, 0.4)	0.00*		0.01*	
θ(3, 0.002) τ(3, 0.004)	0.08*		**1.00**	
θ(3, 0.2) τ(3, 0.004)	0.00*		0.02*	
θ(3, 0.002) τ(3, 0.4)	0.02*		**0.98**	
	**Remaining species and lineages**			
	*I. dugesii* / remaining clades	*I. mexicanus* / remaining clades	*I. punctatus* / remaining clades	*Ictalurus* sp. Nazas / remaining clades
θ(3, 0.2) τ(3, 0.4)	**1.00**	*0.93*	**1.00**	**1.00**
θ(3, 0.002) τ(3, 0.004)	**1.00**	**1.00**	**1.00**	**1.00**
θ(3, 0.2) τ(3, 0.004)	**1.00**	**0.95**	**1.00**	**0.95**
θ(3, 0.002) τ(3, 0.4)	**1.00**	**1.00**	**1.00**	**1.00**

pp values in bold ≥ 0.95 were considered highly supported; pp values in italics ≥ 0.90 and < 0.95 were considered moderately supported; pp values with* < 0.90 were considered weakly supported

In the A11 analyses, almost all recognized species (except *Ictalurus furcatus* and *I. balsanus*) and almost all differentiated populations (except *I.* aff. *lupus* Cuatro Cienegas and *I.* aff. *lupus* Conchos) were supported in at least the same two scenarios as stated above (second (IGθ = 3, 0.002, IG τ_0_ = 3, 0.004) and fourth (IGθ = 3, 0.002, IG τ_0_ = 3, 0.4); Table 3).

**Table 3:** Substitution model and parameters for every locus and sequence matrixes used in the phylogenetic analyses/ species tree

	*cytb*	*cox1*	*atpase8/6*	*RAG1*
**Number of taxa sample**	247/95	203/95	140/95	119/95
**Size (pb)**	1092	609	861	1035
**Variable sites**	456/403	191/173	331/282	141/92
**Informative characters**	345/279	159/129	257/216	53/25
**Model parameters (AICc)**	TPM1uf + G/ GTR + G	K80 + G/ HKY + I + G	TrN + G / TrN + G	TrNef + I/ TrN
**Invariable sites**	--/--	--/0.57	--/--	0.31/--
**Gamma shape**	0.30/0.28	0.17/0.85	0.22/0.23	--/--

## Discussion

This is the first study designed to include all described species of *Ictalurus* (except for *I. meeki*) within a molecular phylogenetic analysis, considering not only the currently recognized and synonymized species [2], but also individuals from well-differentiated populations, regarded as undescribed taxa in several published accounts [2, 17], and from several previously unstudied populations. This could allow improved estimation of the species composition of this genus. Our multilocus and species delimitation approach resolved the phylogenetic relationships among *Ictalurus* species and revealed inconsistencies with respect to the previous taxonomic scheme. Our results also revealed the existence of genetic lineages that could represent undescribed species and corroborated previous synonymies between species proposed on morphological grounds.

### Agreement and conflicts between individual genes, concatenated analysis, and species trees: recovering the evolutionary relationships

Differences among genes were mainly found in the relationships of terminal nodes, which can be attributed to the higher content of variable and informative sites for *cytb* compared to the other mitochondrial genes, *coxI* and *atpase8/6* [28–30], as shown by the number of informative characters and substitution rates. Previous studies found that *coxI* is more conserved than *cytb*, in which more than half of all the amino acid sites were invariable across 250 fish species [31]. Moreover, for the nuclear gene *RAG1*, the lack of resolution in the more derived groups, as was the case with the *pricei* and *lupus* complexes, could be associated with incomplete lineage sorting from this nuclear gene. As evidenced by previous studies, the mitochondrial locus showed a high mutation rate, which is related to its smaller effective population size [32] and is also corroborated by the observed substitution rates.

**Fig. 4:**
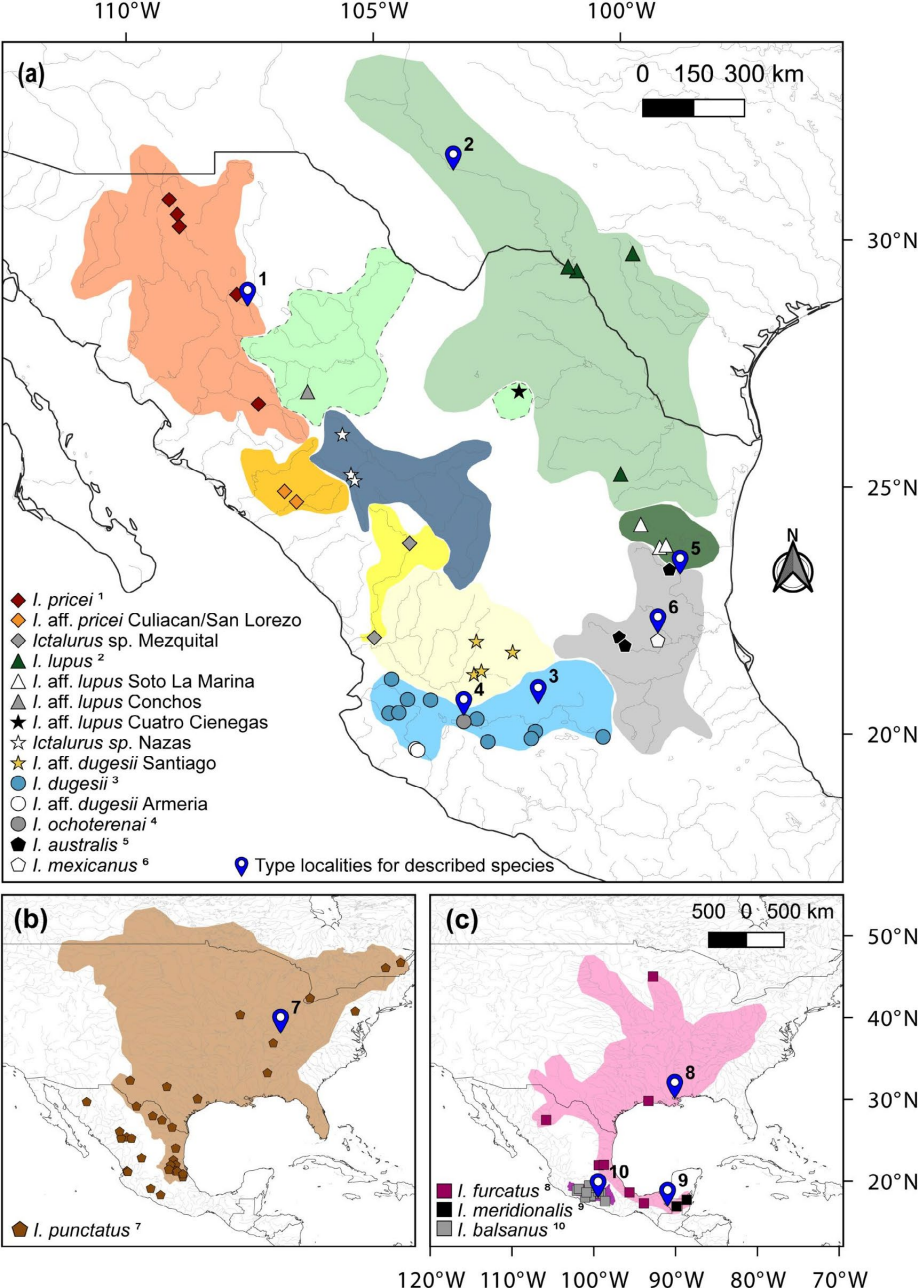
Distribution and sampled locations (type locality was only sampled for a few species) for (a) recognized, synonymized, and putative undescribed species of the genus *Ictalurus*; (b) native and translocated distribution and sampled locations for *Ictalurus punctatus*, and (c) distribution and sampled locations for species of the *furcatus* group. Each colored polygon represents the geographic range of species validated in our molecular study. Gene sequences of ictalurids from Canada and USA localities were obtained from Genbank. All three maps were generated in this study from records for distinct taxa of *Ictalurus*

Although the most informative *cytb* gene placed *I. dugesii* as the sister group of the *pricei* and *lupus* complexes, the concatenated tree, the coalescent species tree, and the *coxI*, *atpase8/6* and *RAG1* genes placed *I. dugesii* as closely related to the *pricei* complex. Nevertheless, the phylogenetic conflict among trees derived from individual genes was present in the low support shown in the species tree. This latter relationship is consistent biogeographically since the western limit of the distribution range of *I. dugesii* (the Chapala-Ameca River) is adjacent to the southern distribution range of the *pricei* complex, which comprises the river basins of the Sierra Madre Occidental, including the Santiago River (Fig. 4). Similar biogeographic patterns have been reported in studies of other co-distributed fish species, including the Goodeids [33], Cyprinids [34] and Catostomids [35]. These freshwater fish groups all feature species that inhabit the central Mexican highlands, the sister species of which are distributed in river drainages of the Sierra Madre Occidental. Previous empirical analyses show that discordances between concatenated and coalescent-based analyses tend to occur when the branches of concatenated analyses are short and weakly supported as a consequence of signal conflict between individual gene trees [36]. Based on this, we regard the species tree as a consistent phylogenetic hypothesis with which to depict the relationships and evolution of the genus *Ictalurus* (Fig. 3).

### Evolutionary relationships and their taxonomic implications

According to the BPP analyses, the second scenario for a small ancestral population and low divergences (IGθ = 3, 0.002, IG τ_0_ = 3, 0.004) and the fourth scenario for a small ancestral population and deep divergences (IGθ = 3, 0.002, IG τ_0_ = 3, 0.4), in the BPP A10 and A11 analyses, support almost all the splits and assignments in the species delimitation, except for *Ictalurus furcatus* and *I. meridionalis*, and the differentiated populations *I.* aff. *lupus* Cuatro Cienegas and *I.* aff. *lupus* Conchos, which were either supported by only one scenario or not supported in either of the analyses (Tables 3 and 4). The results highlight the particular case of *I. balsanus*, which was supported by only one scenario in the A11 analysis, as discussed below. Previous studies, in which different prior parameters were tested, and with values that varied considerably, found unstable support patterns; i.e., higher support for one prior combination and lower support for another [37–39]. Indeed, BPP analyses can be sensitive and potentially misleading, especially regarding the age of the root (τ_0_) that, with increasing values (i.e., 0.04 or 0.4), produced spuriously high posterior probabilities possibly because high values of τ push coalescent events closer to the tips [39]. However, this is not the case in the present study. Despite the increase in τ_0_ (0.004 to 0.4), under the second and fourth scenarios, the supports were unaffected, thus eliminating any misleading results and/or artifacts of the coalescent events. Moreover, there are cases in which θ prior increases considerably (by at least two orders of magnitude), as occurred in the present case (i.e., 0.002–0.2) while speciation probabilities tend to decrease [40]. This is seemingly associated with the use of an extremely high θ, which is not suitable in all cases [37]. Thus, the high values for θ used in scenarios 1 and 3 (0.2) resulted in an unsuitable set of priors for *Ictalurus*. This indicates that scenarios 2 and 4, which supported 13 of the 15 proposed groups as potentially well-delimited taxa, are more suitable for discarding a misleading result.

**Table 4:** Posterior probabilities from all analysis showing the support in the assignments of the species/lineages

**Priors**	***pricei*** **complex**			
	*I. pricei*	*Ictalurus* sp. Mezquital	*I.* cf. *dugesii* Santiago	*I.* cf. *pricei* Culiacan/San Lorenzo
θ(3, 0.2) τ(3, 0.4)	*0.93*	0.07*	0.01*	**1.00**
θ(3, 0.002) τ(3, 0.004)	**1.00**	**1.00**	**1.00**	**1.00**
θ(3, 0.2) τ(3, 0.004)	**0.95**	0.07*	0.04*	**0.97**
θ(3, 0.002) τ(3, 0.4)	**1.00**	**1.00**	**1.00**	**1.00**
	**differentiated lineages of** ***lupus*** **complex**			
	*I. lupus*	*I.* cf. *lupus* Cuatro Cienegas	*I.* cf. *lupus* Conchos	*I.* cf. *lupus* Soto la Marina
θ(3, 0.2) τ(3, 0.4)	0.00*	0.00*	0.02*	**1.00**
θ(3, 0.002) τ(3, 0.004)	**1.00**	**1.00**	*0.90*	*0.90*
θ(3, 0.2) τ(3, 0.004)	0.07*	0.04*	0.02*	0.02*
θ(3, 0.002) τ(3, 0.4)	**0.98**	*0.92*	*0.94*	**1.00**
	***furcatus*** **group**			
	*I. furcatus*	*I. meridionalis*	*I. balsanus*	
θ(3, 0.2) τ(3, 0.4)	0.00*	0.00*	0.00*	
θ(3, 0.002) τ(3, 0.004)	**1.00**	**1.00**	**1.00**	
θ(3, 0.2) τ(3, 0.004)	0.13*	*0.90*	0.21*	
θ(3, 0.002) τ(3, 0.4)	0.00*	0.00*	0.00*	
	**Remaining species and lineages**			
	*I. dugesii*	*I. mexicanus*	*I. punctatus*	*Ictalurus* sp. Nazas
θ(3, 0.2) τ(3, 0.4)	**1.00**	*0.93*	**1.00**	**1.00**
θ(3, 0.002) τ(3, 0.004)	**1.00**	**1.00**	**1.00**	**1.00**
θ(3, 0.2) τ(3, 0.004)	**1.00**	**0.95**	**1.00**	**0.95**
θ(3, 0.002) τ(3, 0.4)	**1.00**	**1.00**	**1.00**	**1.00**

pp values in bold ≥ 0.95 were considered highly supported; pp values in italics ≥ 0.90 and < 0.95 were considered moderately supported; pp values with* < 0.90 were considered weakly supported

As with the earlier phylogenetic hypothesis based on morphology [2], a dichotomy between the *furcatus* and *punctatus* groups was found in our analyses. Interestingly, our study revealed several differences in species relationships when compared with the most recent phylogenetic hypothesis involving catfishes of the family Ictaluridae, in which the molecular data and morphological characters of extant and fossil species were used [11].

#### The furcatus group

The results of the species delimitation analyses do not support the split of *Ictalurus meridionalis* and *I. furcatus* in the A10 analysis, and the assignments in the A11 analysis for both of these taxa were highly supported only by the second BPP scenario and, in the case of *I. meridionalis*, only moderately supported by the third scenario. It is therefore not possible to validate these two taxa and, based on the principle of priority, *I. meridionalis* is considered a synonym of *I. furcatus*, corroborating its status as a synonymized species as previously stated by Lundberg [2]. This is consistent with the fact that only *coxI* supported the reciprocal monophyly (Fig. S1) and that values of genetic divergence of 1.5% were found with *cytb* between *I. meridionalis* (Hondo River and Peten samples) and *I. furcatus* from across a wide geographic range (samples from the Mississippi to Coatzacoalcos). This divergence value lies below the minimum inter-species level within Ictaluridae (1.8-3.6%) [15, 25, 26, 41], and these results indicate that the morphological differentiation reported [42] could be the result of geographical variation within the widespread *I. furcatus*. Although the geographic coverage for *I. furcatus* is incomplete, the samples collected from the extremes of the distribution range of the species (from the Mississippi River in the USA to the Hondo River in Belize) presented two structured populations. Therefore, a future phylogeographic study including optimal sampling along the distribution range of the species is necessary to determine the genetic pattern of these two divergent groups.

Unexpectedly, the assignment of the species *Ictalurus balsanus*, which is one of those that diverged early within the genus, was only supported by the second scenario in the A11 analysis. However, the deep divergence level of this taxon strongly validates it as a recognized species. Based on the results presented herein, we consider the *furcatus* group to be formed by the species *I. balsanus* and *I. furcatus*, as was originally established [2], pending a phylogeographic study with a wider geographic range for *I. furcatus*.

Our results do not entirely support the previous phylogenetic hypothesis [13], which included molecular data and morphological characters as a total evidence analysis. In this study [13], *I. balsanus* was found to be the early divergent species and the sister species of the clade containing all other living and fossil species within the genus. This discrepancy might have been the result of one of the following: the possible influence of the different phylogenetic signals from the genes used (*16s*, *12s*, and *RAG2*, which were not used in the present study) or the methodological implications associated with the considerable amount of missing data, particularly the lack of complete molecular data for most taxa in the analysis by Arce-H et al. [13]. For instance, only two of the five genes were included in that analysis for *I. balsanus*. Moreover, it is known that a large quantity of missing data, together with high rates of change, can lead to long-branch attraction [43]. This occurs more frequently using Maximum Parsimony than probabilistic methods [44] such as those used in Arce-H et al. [13].

#### The punctatus group

The *punctatus* group included the five remaining recognized species of *Ictalurus* (*I. pricei*, *I. lupus*, *I. dugesii*, *I. mexicanus*, and *I. punctatus*) and five well-differentiated populations (*Ictalurus* sp. Santiago, *Ictalurus* sp. Mezquital, *Ictalurus* sp. Culiacan/San Lorenzo, *Ictalurus* sp. Conchos/Cuatro Cienegas, and *Ictalurus* sp. Nazas), four of which were previously recognized as undescribed species [2, 17, 19], and the other is a new well-differentiated population revealed in the present study (*Ictalurus* sp. Soto la Marina). All 11 of these groups were supported by BPP analyses (Tables 3 and 4).

*Ictalurus punctatus*: The channel catfish presents a native distribution stretching from the north in the Great Lakes, southern Canada, Hudson Bay (Red River drainage), and the Missouri-Mississippi River basins, extending southwards to the Panuco River on the Mexican Atlantic slope [17, 19, 45]. Although the present study lacks broad geographic coverage for *I. punctatus*, we included samples from both nearby (e.g., individuals from the same locality, Lake Michigan or the Grande River) and distant (e.g., individuals from Canada and the Mississippi to the Panuco River in Mexico) geographical points. This allowed us to cover the genetic variation and differentiation that exists within this species, and to confirm its phylogenetic position within the genus. All the samples identified as *I. punctatus* (excluding the specimens involved in the mt/nuc discordance) were recovered as a monophyletic assemblage in the concatenated and species tree analyses. In addition, low genetic differentiation between nearby or distant geographic samples was detected with all genes, indicating that all these populations should be treated as a single genetic group or species. Interestingly, a similar pattern was uncovered when dealing with a specific parasite of channel catfishes [46]. The trematode *Phyllodistomum lacustri* Loewen, 1929, which occurs in the urinary bladder of its hosts, was genetically identical in channel catfishes sampled in Canada, the USA, and northern Mexico [46]. However, a cryptic species complex of *P. lacustri* was uncovered in populations of catfishes occurring in several river basins of Mexico, where *Ictalurus* has experienced a great diversification process [46].

All the specimens of channel catfish sampled were morphologically identified or genotyped (with mitochondrial genes) as *I. punctatus*; however, we found four cases of mt/nuc discordance in the phylogenetic position, that involved specimens from the Santa Maria River in the Panuco River basin, and the Pantepec River in the Tuxpan River basin. These were assigned to the *I. mexicanus* clade with the nuclear locus, and a specimen from the Sabinas River within the Bravo River basin was assigned as part of the *lupus* complex with the nuclear locus.

Due to the high genetic divergence values between *I. punctatus* and the *I. mexicanus* – *lupus* complex (7.5% based on *cytb* in the former two, and 7.8–8.7% in the latter), as well as the phylogenetic position with both mtDNA and nDNA of most samples from the *I. mexicanus* – *lupus* complex, the possibility of an incomplete lineage sorting can be discarded. Another plausible explanation could be the occurrence of a hybridization event. However, a morphological revision of adult individuals would be necessary [47], as well as an extensive study with nuclear loci, to obtain, for example, heterozygous individuals with nuclear loci, indicating the presence of one allele of each taxon, as has been found for other native species; e.g., *I. pricei* and *I. lupus vs. I. punctatus* by Gutierrez-Barragán et al. [48].

*Ictalurus australis* and *I. mexicanus*: Both species were described by Meek [49], the type locality of *I. australis* is the Forlon River, a tributary of the Guayalejo River sub-basin of the Panuco River Basin, and its distribution extends along the Panuco, Tuxpan, Cazones, Tecolutla and Nautla Rivers [50], reaching the Blanco River within the Papaloapan River basin [51]. The type locality of *I. mexicanus* is the Gallinas River, and it is considered endemic to this tributary in the upper Panuco (17, 52–53). The taxonomic status of *I. australis* has been questioned and the species has even been considered a junior synonym of *I. punctatus* (2, 54–55), although several studies still consider *I. australis* as a valid taxon (19, 56–57). However, according to Miller et al. [17], *I. mexicanus* is restricted to the Gallinas River and the populations in the Panuco basin are considered a closely related but undetermined taxon.

We included specimens from the type locality for both species and, according to the low genetic divergence shown between them (< 0.5% with the mitochondrial *cytb* gene) and the lack of reciprocal monophyly within *Ictalurus mexicanus/australis* with the *cytb* and *coxI* genes, we consider that the samples of *I. mexicanus* from Gallinas River up- and downstream of the Tamul waterfall, those distributed along the Verde River, and those from the Guayalejo River (according to *RAG1* gene) do not represent two distinct lineages, suggesting that *I. mexicanus, I. australis* and/or *Ictalurus* sp. *sensu* Miller et al. [17] are part of the same taxonomic unit. Thus, based on the principle of priority, and pending the inclusion of more samples from across the distribution range of this taxon and integrative taxonomic analyses, we propose recognition of this population as *I. mexicanus*. The lack of *I. australis* samples from the southern basins such as the Tuxpan, Cazones, Tecolutla, Papaloapan, and Nautla Rivers (50–51) indicates the need to include samples from the entire potential distribution range in a phylogeographic analysis to explore the relationship with, and patterns of differentiation from, *I. mexicanus* of the Panuco basin.

*The pricei complex: northwestern pacific group*: This monophyletic group is represented by one recognized species, *Ictalurus pricei*, and three well-differentiated populations with a divergence of 1.8–1.9% at the *cytb* locus. This value exceeds the minimum divergence found between well-recognized species within Ictaluridae (15, 29–30). These four divergent groups were corroborated by the BPP species delimitation test as independent taxonomic units. The northernmost taxon corresponds to *I. pricei sensu stricto* from the Yaqui and Fuerte rivers. We did not include samples from other basins such as the Sonora, Mayo, and Casas Grandes Rivers [17, 58], and these populations therefore remain to be analyzed to determine whether or not they can be nested with *I. pricei* from the Yaqui and Fuerte rivers.

Another differentiated group was represented by the specimens of *Ictalurus* sp. from the Culiacan/San Lorenzo rivers, confirming previous morphological findings that indicated this population as a potential undescribed taxon [20]. Similarly, the specimens of *Ictalurus* sp. from the Mezquital River were corroborated as representing a potentially undescribed taxon in our analyses, as previously recognized by other authors (2, 18–19). Finally, the differentiated population of *Ictalurus* sp. from the Santiago River, which corresponds to the southernmost independent taxonomic unit of the *pricei* complex, was also corroborated as a potentially undescribed taxon, originally referred to by Miller et al. [17] as an undescribed species related to *I. dugesii*. The results of a study by Rosas-Valdez et al. [46] on the recognition of a cryptic species complex of the trematode *Phyllodistomum lacustri* may lend further support. The populations of the parasite sampled in catfishes of the Mezquital River basin (identified by the authors as *Ictalurus* sp.), and those from the Lerma River basin at San Juanico Dam (identified as *I. dugesii*), but also including introduced specimens of *I. punctatus*, were retrieved as cryptic species in the molecular phylogenetic analyses of two genes (*28s* and *coxI*), with each representing a separate species.

*Ictalurus dugesii*: The *Ictalurus* samples from the Lerma-Chapala system, including specimens identified as *I. ochoterenai* from Chapala Lake and the populations from the Ameca and Armeria River basins, were nested within the *I. dugesii* clade. Regarding *I. ochoterenai* and *I. dugesii*, we found a lack of reciprocal monophyly, as well as a genetic divergence (< 0.5%) below the minimum limit recorded for Ictaluridae using *cytb* (1.8–3.6%) [15, 25, 26, 41]. Based on the above, the present study confirms *I. ochoterenai* as a synonym of *I. dugesii*, as proposed by other authors [2, 59].

An undescribed form of *I. dugesii* was proposed by Miller et al. [17], corresponding to the populations of *I. dugesii* from the Armeria River. In this case, even though we found reciprocal monophyly with *cytb*, this group was nested within *I. dugesii* when data were analyzed using the other two mitochondrial genes. It also presented low genetic divergence (0.2%), which suggests a relatively recent isolation event within the Armeria River basin. Thus, the distribution of *I. dugesii* is confirmed for three disjunct river basins in central Mexico, i.e., the Lerma-Chapala system and the Ameca and Armeria Rivers. This is in accordance with other co-distributed fish species in the region, such as the catostomid *Moxostoma austrinum* Bean, 1880 [60], and several species of goodeids (61–62).

*The lupus complex*: The distribution range of the headwater catfish, *I. lupus* comprises several rivers of the Gulf of Mexico slope, including the Colorado, Guadalupe, and Nueces drainages in Texas, the Bravo River drainage in the United States and Mexico, as well as the San Fernando, Soto la Marina, and Conchos Rivers, and the endorheic Cuatro Cienegas basin in Coahuila, Mexico [47]. For the *cytb* analyses, *I. lupus sensu stricto* from the Pecos and Devil Rivers presented a relatively high genetic divergence with respect to the other populations (*Ictalurus* sp. Conchos/Cuatro Cienegas, and *Ictalurus* sp. Soto la Marina), ranging from 2 to 2.8%. Although *Ictalurus* sp. Conchos and *Ictalurus* sp. Cuatro Cienegas presented a high divergence value (1.9% in the *cytb* gene), and the species delimitation test resolved only three groups as potentially independent taxonomic units: *I. lupus*, *Ictalurus* sp. Conchos/Cuatro Cienegas, and *Ictalurus* sp. Soto la Marina (Tables 3 and 4).

The genetically differentiated *Ictalurus* sp. Conchos seems to correspond to the previously reported divergent population of *Ictalurus* from the Conchos River, which also occurs in sympatry with *I. lupus* in the lower Bravo River (2, 17–18, 54). In addition, *Ictalurus* sp. Soto la Marina was supported as a candidate species in the BPP analyses and presented the highest genetic divergence within the *lupus* complex, merging as a novel recognized independent evolutionary lineage and putative species. According to our findings, an integrative taxonomic study with broader geographic coverage of *I. lupus*, as well as a detailed study of the morphoanatomy of this species, are required to test the possible taxonomic independence of the three divergent evolutionary units within the *lupus* complex: *I. lupus sensu stricto*, *Ictalurus* sp. from the Conchos River and Cuatro Cienegas valley, and *Ictalurus* sp. from the Soto la Marina River.

*Ictalurus* sp. Nazas: Our study resolved the specimens of *Ictalurus* from the Nazas River as a highly divergent group within the *punctatus* complex. All the analyses confirmed this population as a reciprocally monophyletic group, which was apparently the first to diverge within the central-western drainages, indicating this as a potential taxon in the BPP analyses. Our results support previous studies that consider the population of *Ictalurus* from the Nazas as an undescribed species, although these studies provide no details regarding the morphological characters that distinguish this from other congeners [2, 18, 19, 63] and, consequently, a description of the new species is still pending. Other studies indicate that the Nazas River is an area where independent genetic lineages of fishes occur [64–68].

### Divergence times and evolution of ***Ictalurus***

The genus *Ictalurus* is confirmed as an ancient group of fishes; our dating analysis placed the earliest split between the *punctatus* and *furcatus* groups at ca. 33 − 23 Mya (Fig. 3), which is partially consistent with previous studies that estimate the age of the crown group of *Ictalurus* at 38 − 20 Mya [69] and 37 − 30 Mya [13]. The considerable species richness in the fossil record, together with the inferred divergence times, indicate that the genus has experienced several extinction events during its evolutionary history, particularly in the case of early-diverging species. The most abundant fossil record for *Ictalurus* has been found in North America (13, 70–71), and includes the oldest fossil, *Ictalurus rhaeas* Cope, 1891, discovered at the Cypress Hill Oligocenean Formation, located in southern Saskatchewan, Canada. Hydrographically, this formation is included in the Missouri-Mississippi drainage system [70]. The region originated in the Late Eocene and is one of the most important in terms of explaining the evolution of the North American freshwater fish fauna, including ancient fish fauna elements such as gars (Lepisosteidae), bowfin (Amiidae), and mooneyes (Hiodontidae), as well as the most recent representatives of the North American fish fauna, such as catostomids, salmonids, percids, cyprinids, and catfishes [72]. The first lineages, derived during the evolution of the genus *Ictalurus* in the early Miocene, show high dispersal capacity and an adaptation to the environmental conditions that allowed them to expand their distribution range along the Atlantic slope of North America, as reflected in the widespread distribution of *I. furcatus* and *I. punctatus*, from southern Canada through the US and Mexico southwards to Belize, including the dispersal of the ancestor of *I. balsanus* to the Balsas River on the Pacific slope of Mexico.

The considerable diversification observed in the *punctatus* group shows a first split with the cladogenetic event of *I. mexicanus* in certain tributaries of the Panuco River basin. Although no explanations have been proposed for the origin and isolation of this species in the Verde or Gallinas rivers (currently inhabited by *I. mexicanus*), this region has served as an area of speciation for different groups, with the occurrence of a significant number of endemic species of cyprinids [65], goodeids [61], cichlids (73–74) and poecilids [17]. This high level of endemism seems to be related to the intense volcanism of the Miocene-Pliocene, which promoted sudden subsidence of the graben structure in the basin and the formation of shallow lakes [75]. *Ictalurus mexicanus* followed the same speciation pattern as other fish species in the area.

The next diversification event was the cladogenesis of *Ictalurus* sp. Nazas, and the *lupus* complex distributed in the Northern basins of the Bravo and Soto la Marina Rivers, followed by the formation of the *I. dugesii* and the *pricei* complex with wide distribution along the Pacific slope drainages, from the Yaqui River in Sonora southwards to the Lerma River in Michoacan and Guanajuato. Several fish groups experienced the same speciation pattern, including the cyprinids and catostomids [35, 65]. These speciation events associated with the Bravo, Nazas, and Northern pacific slope drainages have been explained by hypothetical connections along the extensive paleo-hydrological system in the Chihuahuan desert region, which dates back to the Oligocene and included the Conchos River, the main channel of the Bravo River, the Nazas River basin, and the headwaters of several western Pacific river drainages (i.e., the Yaqui and Mezquital Rivers) [66], and the isolation of which is estimated at ca. 5 Mya [18]. The biogeographic role of the Chihuahuan desert paleo-hydrological system is closely associated with the tectonic activity of the Bravo River rift, together with the arid conditions that have been prevalent since the Miocene [75]. This paleo-hydrological system could have had two main roles in the evolution of the genus *Ictalurus*, in a similar manner to that reported for other fish groups (60, 64, 66–67, 76–78): *(1)* most of the diversification events occurred within or were promoted across the region, and *(2)* the region acted as a corridor for the *punctatus* group, allowing them to colonize the Pacific slope, where they subsequently diversified.

In general, derived from a temporary and geographically extensive evolutionary history, most of the speciation events within the genus *Ictalurus* occurred in the river basins of Mexico. This implies an increase in both species richness and levels of endemism. The results of our study pose a huge challenge for research on the diversity and conservation of this representative and important group of North American freshwater fishes. The most recent evaluation of the IUCN red list for fish species in Mexico categorizes only *I. pricei* and *I. mexicanus* in the categories “Endangered” and “Vulnerable”, respectively [79], mainly based on the premise of the high distribution range of most of the species. However, our research contradicts this notion of the wide distribution of the species and raises the need for a more detailed conservation evaluation of the independent evolutionary units or undescribed species found in the present study to adequately protect the true diversity of the genus *Ictalurus* in Mexico.

Finally, parasitological data is frequently used, along with the phylogenetic history of hosts, to explain concurrent patterns. In this context, the members of the *punctatus* group were typical hosts of the trematode *Phyllodistomum lacustri*, a parasite of the urinary bladder of several species of ictalurids [46]. Molecular phylogenetic analyses of specimens of *P. lacustri* from *Ictalurus punctatus* sampled from its natural distribution range across Canada, the USA, and northern Mexico show a pattern congruent with that of the hosts [46]. Interestingly, the presence of a cryptic species complex of *P. lacustri* that includes other species of *Ictalurus*, such as *I. dugesii* in the Santiago basin, and *I. pricei* (*Ictalurus* sp. Mezquital in this study) from the Mezquital basin [46] was also reported. These river basins of western Mexico appear to be important areas for the diversification of *I. dugesii* and *I. pricei* and their parasites. We believe that future studies aimed at uncovering diversification patterns of freshwater fishes should, whenever possible, utilize other information sources to corroborate hypotheses. We also consider that the history of the host-parasite association could constitute a robust proxy with which to test such patterns of diversification.

## Conclusions

The results obtained in the present study of the systematics, biogeography, and evolution of the genus *Ictalurus* are based on the most extensive and comprehensive taxonomic sampling to date. Despite the conflicting phylogenetic signals from individual genes, the results of this study conducted using several approaches, including gene trees, a concatenated dataset of nuclear and mitochondrial genes through a multi-locus approach, and the coalescent species tree, recovered a complementary signal, and thus yielded a plausible phylogenetic hypothesis for the genus. Moreover, our study supports the recognition of 13 taxonomically independent units, including seven previously recognized species, *I. punctatus*, *I. mexicanus*, *I. dugesii*, *I. lupus*, *I. pricei*, *I. furcatus*, and *I. balsanus*, and six putative undescribed species, *Ictalurus* sp. Culiacan/San Lorenzo, *Ictalurus* sp. Mezquital, *Ictalurus* sp. Santiago, *Ictalurus* sp. Conchos/Cuatro Cienegas, *Ictalurus* sp. Soto la Marina, and *Ictalurus* sp. Nazas. These independent evolutionary units require a detailed morphoanatomical study to be formally described as new ictalurid species. We also corroborated previously synonymized species such as *I. ochoterenai, I. australis*, and *I. meridionalis*. As in other taxonomically underestimated fish groups (35, 62, 65, 78–79), there has been a lack of morphological studies and/or taxonomic revisions addressing these differentiation patterns in Ictaluridae, a conspicuous, economically important, popular, and important group of fishes. An integrative taxonomy approach is required to describe the evolutionary history of the group and to achieve a better understanding of its species composition and thereby improve its conservation.

## Materials and methods

### Sampling and sequencing

A total of 187 individuals of the genus *Ictalurus* were sampled, including members of all recognized species, according to Lundberg [2] and Miller et al. [17]. Sampling also included several populations recognized as undescribed taxa (2, 17–18, 20), other poorly studied populations, and three previously synonymized species [2] (Fig. 4; Table 5 and S1). Most of the samples were obtained from three tissue banks: Colección de Peces de la Universidad Michoacana de San Nicolás de Hidalgo, Mexico (CPUM_SEMARNAT-Mich-PEC-227-07-09), Colección Ictiológica del Museo Nacional de Ciencias Naturales, España (MNCN_ICTIO), and Colección de Tejidos del Laboratorio de Genética de la Conservación, CIBNOR, Mexico (LGC_ADN) (Table S1). Other tissue samples were donated by fishermen.

**Table 5:** Taxonomic sampling of the genus *Ictalurus* used in the present study

Recognized species	Species previously synonymized	Differentiated populations (Putative undescribed species)
*Ictalurus lupus*, Girard 1858		*I.* cf. *lupus* Conchos
		*I.* cf. *lupus* Cuatro Cienegas
		*I.* cf. *lupus* Soto la Marina*
*Ictalurus pricei*, Rutter 1826		*I.* cf. *pricei* Culiacan/San Lorenzo
*Ictalurus dugesii*, Bean 1880	*Ictalurus ochoterenai*, Bean 1880	*I.* cf. *dugesii* Armeria
		*I.* cf. *dugesii* Santiago
*Ictalurus mexicanus*, Meek 1904		*Ictalurus* sp. Mezquital
*Ictalurus punctatus*, Rafinesque 1818	*Ictalurus australis*, Meek 1904	*Ictalurus* sp. Nazas
*Ictalurus balsanus*, Jordan & Snyder 1900		
*Ictalurus furcatus*, Le Suer 1840	*Ictalurus meridionalis*, Gunther 1864	

Present study included, currently recognized species (*sensu* Lundberg, 1992 and Miller et al., 2005), species previously synonymized

(Lundberg, 1992), undescribed species (*sensu* Lundberg, 1992; Miller et al., 2005; Ruíz-Campos et al., 2020), and * a new well

-differentiated population revealed in this study

Isolation of genomic DNA was performed using the QIAGEN BioSprint Dneasy Tissue and Blood Kit (Qiagen, Valencia, Ca, USA), following the manufacturer’s instructions. The mitochondrial genes Cytochrome b (*cytb*), Cytochrome oxidase subunit 1 (*coxI*), and ATP synthase 6 and 8 (*atpase8/6*) were amplified using the primers GludG [80] and H16460 [81], FISHF1 and FISHR1 [82], COIII.2, and ATP8.2 [83], respectively. The nuclear recombination-activation 1 gene (*RAG1*) was amplified using the primers RAG1F and RAG9R [84]. Amplifications were conducted in a final reaction volume of 25 µl, comprising: 50–100 ng genomic DNA, 1x PCR buffer (containing 1.5mM of MgCl_2_), 0.2 mM of each dNTP, 0.5 µM of each primer and 1 U of Taq DNA polymerase (Invitrogen). The thermocycler parameters for all amplifications consisted of an initial 2 min denaturation step at 94 ºC. Subsequent cycling parameters for each region were as follows: For *cytb*, 35 cycles of 45 s at 94 ºC, 60 s at 48 ºC and 90 s at 72 ºC, with a final extension step of 5 min at 72 ºC; for *coxI*, 35 cycles of 30 s at 94 ºC, 30 s at 52 ºC and 60 s at 72 ºC, with a final extension step of 10 min at 72 ºC; for *atpase8/6*, 35 cycles of 45 s at 94 ºC, 60 s at 48 ºC and 90 s at 72 ºC, with a final extension step of 5 min at 72 ºC; and for *RAG1*, 35 cycles of 30 min at 95 ºC, 45 s at 56 ºC and 90 s at 72 ºC, with a final extension step of 7 min at 72 ºC. Positive amplicons were purified using the ExoSAP-IT PCR Product Cleanup Reagent. Sequencing was performed by the Macrogen Korea sequencing service. Sequences were manually aligned in Mega v7.0 [85]. *RAG1* alleles were separated using the PHASE algorithm [86], as implemented in DnaSP v. 6.10.01 [87]. The datasets generated during the current study are available in Genbank (accession numbers ON008558-ON023996). Taxon names and voucher information and associated Genbank numbers are presented in an additional file in Table S1. Moreover, additional sequence data for *cytb*, *coxI, atpase8/6*, and *RAG1* of the ingroup and the functional outgroups *Cranoglanis bouderius* Richardson, 1846 and *Pangasianodon hypophthalmus* Sauvage, 1878 were retrieved from Genbank (Table S1).

### Phylogenetic analyses and sequence divergence

We obtained the best-fitting models of nucleotide substitution for each locus (Table 4) considering the Akaike information criteria (AIC) in the program jModelTest2 (88–89). Substitution saturation for the three codon positions at all four loci was assessed with Xia´s method (23–24) using DAMBE 7.3.32 [90], which estimates an entropy-based index of substitution. To infer the phylogenetic relationships, we conducted a Bayesian Inference (BI) using MrBayes 3.2.3 [91]. We used four simultaneous and independent runs consisting of four chains for 10 million generations, with trees sampled every 1000 generations to calculate posterior probabilities (PP). Convergence and stationary distribution of the runs were verified by the average standard deviation of the split (ASDSF < 0.01) and with a potential scale reduction factor (PSRF) close to 1.0 for all parameters, as indicated in MrBayes. The effective sample size (ESS) for all the parameters was > 200, as visualized in Tracer 1.5 [92]. The first 10% of generations were removed as burn-in. We also performed a Maximum Likelihood (ML) analysis on RaXML v.7.2.8, implementing a GTRCAT model with 1000 bootstrap replicates [93–95]. The findings for the best-fitting model of nucleotide substitution and the two phylogenetic reconstruction approaches were run in the CIPRES Science Gateway V.3.3 portal [96]. Both probabilistic methods were run for all four individual loci, *cytb*, *coxI, atpase8/6*, and *RAG1*, as well as for a concatenated matrix including all four loci. Two important points should be noted: firstly, due to the lack of at least two loci for *I. lupus* and *I.* aff. *pricei* Culiacan/San Lorenzo (Table S1), these genetically differentiated groups were excluded from the concatenated matrix, and secondly, based on the findings of reticulate relationships (see results), several individuals morphologically determined as *I. australis* and *I. punctatus* (Table S1) were also excluded from the concatenated analyses. Sequence divergences among all recognized, synonymized and undescribed species (Table 5) were estimated using the mean among-groups uncorrected (*p*) genetic divergences (Table S3-S6) in MEGA 7.

### Coalescent-based species tree and divergence time estimation

Using the four genes concatenated matrix (Table 4), we conducted a multispecies coalescent analysis for 95 individuals, representing the six recognized species (except *I. lupus*), one synonymized species (*I. meridionalis*), and five differentiated populations (*I.* aff. *lupus* Conchos, *I.* aff. *lupus* Cuatro Cienegas, *I.* aff. *dugesii* Santiago, *Ictalurus* sp. Mezquital, and *Ictalurus* sp. Nazas), all recovered as monophyletic groups in the concatenated analysis and presenting a higher *cytb* genetic divergence than that found between well-recognized species of ictalurids (1.8 − 3.6%) [15, 25, 26, 41]. The synonymized *I. meridionalis*, which presented a genetic divergence of Dp = 1.5% (a value close to the minimum limit), was also included as an independent taxon in the analysis. Moreover, *I. ochoterenai* and *I. australis*, with Dp < 0.6, which is lower than that of their closest relatives *I. dugesii* and *I. mexicanus*, were excluded. For this analysis, we used *Ameiurus natalis* Lesueur, 1891 as an outgroup in *BEAST [97] implemented in the software Beast v.1.8.3 [98]. To calibrate the species tree, we performed a time-calibrated tree with four fossil constraints, which allowed us to obtain the substitution mean rates for all four loci for subsequent use in the species tree, applying an uncorrelated lognormal relaxed clock prior (see Appendix S2). For species tree priors and population size models, a speciation Yule process and a constant species tree population size were chosen. We performed four independent MCMC runs for 300 million generations, sampling every 3000 generations. Chains convergence was assessed by visualizing the sampled parameter values in Tracer 1.5. We discarded 10% of the generations as burn-in and pooled the estimated parameters using the Log-Combiner module in the BEAST package. The maximum clade credibility species tree was obtained from the Tree-Annotator module in the BEAST package.

### Species delimitation analysis

A Bayesian multi-locus species delimitation analysis was conducted using the program Bayesian Phylogenetics and Phylogeography (BPP) v3.4 [99]. In the first approach, an A10 analysis (species delimitation test using a user-specified guide tree [100–102] was performed. This method uses a multispecies coalescent model to compare the posterior probabilities of different species delimitation models (100–101) and accommodates lineage sorting due to ancestral polymorphisms [100]. Given that it was possible to perform the analysis with unequal taxon sampling for some loci, the species *I. lupus* and *I.* aff. *pricei* Culiacan/San Lorenzo, which lack the *atpase8/6* and *RAG1*, were included. The topology based on *cytb* was used as a user-specified guide tree with which to conduct the species delimitation, enabling us to assess all seven recognized species (Table 5), one synonymized species (*I. meridionalis*), and six differentiated populations (*I.* aff. *lupus* Conchos, *I.* aff. *lupus* Cuatro Cienegas, *I.* aff. *dugesii* Santiago, *Ictalurus* sp. Mezquital, *Ictalurus* sp. Nazas, and *I.* aff. *pricei* Culiacan/San Lorenzo). Moreover, to validate the outcomes of the A10 analysis of species delimitation, we also performed the A11 analysis (a joint comparison of species delimitation/assignment and species tree estimation) [28, 102] using the same set of priors. Following the strategy of Chan and Grismer [39] to assess the species delimitation/assignment using A11, the present study conducted this analysis independently in four main groups: the *pricei* complex, the *lupus* complex, the *punctatus* group, and the *furcatus* group.

The following parameter sets were specified for both the A10 and A11 analyses: a fixed guide tree, a Dirichlet distribution (α = 2) to account for variation in mutation rates among loci and to discern how the effective ancestral population size and the divergence influenced the results, and an inverse-gamma prior (IG) used to specify the population size parameter θ and root-age τ_0_ of the species tree considering four different scenarios. These used α = 3 for a diffuse prior and adjusted the β to cover the different alternative scenarios: *(1)* large ancestral population sizes and deep divergences (IGθ = 3, 0.2, IG τ_0_ = 3, 0.4); *(2)* small ancestral population sizes and shallow divergences (IGθ = 3, 0.002, IG τ_0_ = 3, 0.004); *(3)* large ancestral population sizes and shallow divergences (IGθ = 3, 0.2, IG τ_0_ = 3, 0.004), and *(4)* small ancestral population sizes and deep divergences (IGθ = 3, 0.002, IG τ_0_ = 3, 3, 0.4). Reversible jump (rj) MCMC was run for 500,000 generations, with a burn-in of 8000 and a sampling frequency of two. To assess convergence, we performed the analyses three times to confirm consistency between runs. Posterior probabilities (pp) ≥ 0.95 were considered highly supported, pp ≥ 0.90 and < 0.95 were considered moderately supported, and pp < 0.90 were considered weakly supported.

### Electronic supplementary material

Below is the link to the electronic supplementary material.

Supplementary Material 1

## Data Availability

The datasets used and/or analyzed during the current study are available in the GenBank-NCBI database under the accession numbers ON008558-ON009000, ON009002 and ON023863- ON023993, ON023996.
