# Supplementary material for: Multi-locus phylogeny of the catfish genus Ictalurus Rafinesque, 1820 (Actinopterygii, Siluriformes) and its systematic and evolutionary implications
Source: BMC Ecol Evol. 2023 Jun 28;23:27. doi: 10.1186/s12862-023-02134-w (PMC10304232; doi:10.1186/s12862-023-02134-w)

**Table S1.** Geographic distribution range, geographic coordinates, voucher specimens, and the Genbank accession number of analyzed sequences. *Hybrid individuals and their respective crosses suggested by genetic data are shown in the voucher tissue column.

| **Taxa** | **Locality** | **Voucher tissue** | ***cytb*** | ***cox1*** | ***atpase8/6*** | ***RAG1*** |
| --- | --- | --- | --- | --- | --- | --- |
| *Ictalurus* sp. Mezquital | 1. Tunal River at Melones bridge, | CPUM-1790 | ON008811 | ON023863 | ON008558 | -- |
|  | Nombre de Dios town; Mezquital | CPUM-1791 | ON008812 | ON023864 | ON008559 | ON008696 |
|  | basin, Durango, Mex. | CPUM-1792 | ON008813 | ON023865 | ON008560 | ON008697 |
|  |  | CPUM-1794 | ON008814 | ON023866 | ON008561 | ON008698 |
|  |  | CPUM-4988 | ON008815 | ON023867 | ON008562 | ON008699 |
|  |  | CPUM-4989 | ON008816 | -- | -- | -- |
|  |  | CPUM-4990 | ON008817 | -- | -- | -- |
|  |  | CPUM-4991 | ON008818 | -- | -- | -- |
|  |  | CPUM-4992 | ON008819 | -- | -- | -- |
|  |  | CPUM-4993 | ON008820 | ON023868 | ON008563 | ON008700 |
|  |  |  |  |  |  |  |
|  | 2. Mezquital River at west of Ruiz | CPUM-5133 | ON008821 | -- | -- | -- |
|  | town; Mezquital basin, Durango, | CPUM-5134 | ON008822 | -- | -- | -- |
|  | Mex. |  |  |  |  |  |
|  |  |  |  |  |  |  |
|  | 3. Mamey River, 11 km northwest | CPUM-5156 | ON008823 | ON023869 | ON008564 | ON008701 |
|  | of the Mecatan town | CPUM-5157 | ON008824 | ON023870 | ON008565 | -- |
|  |  | CPUM-5158 | ON008825 | ON023871 | ON008566 | ON008702 |
|  |  |  |  |  |  |  |
| *Ictalurus* cf. *dugesii* Santiago | 4. Verde River at Belen del | CPUM-1744 | ON008826 | ON023872 | ON008567 | -- |
|  | Refugio town; Verde basin, | CPUM-5898 | ON008827 | -- | -- | -- |
|  | Jalisco, Mex. | CPUM-5899 | ON008828 | ON023873 | ON008569 | ON008703 |
|  |  | CPUM-5900 | ON008829 | ON023874 | ON008570 | -- |
|  |  | CPUM-5902 | ON008830 | ON023875 | ON008571 | -- |
|  |  | CPUM-5904 | ON008831 | ON023876 | ON008572 | -- |
|  |  | CPUM-5905 | ON008832 | -- | -- | -- |
|  |  |  |  |  |  |  |
|  | 5. Juchipila River at Jalpa Town; | CPUM-5791 | ON008833 | ON023877 | ON008573 | ON008704 |
|  | Juchipila basin, Zacatecas, Mex. | CPUM-5792 | ON008834 | -- | -- | -- |
|  |  | CPUM-5793 | ON008835 | -- | -- | -- |
|  |  | CPUM-5794 | ON008836 | ON023878 | ON008574 | -- |
|  |  | CPUM-5795 | ON008837 | -- | -- | -- |
|  |  | CPUM-5796 | ON008838 | -- | -- | -- |
|  |  |  |  |  |  |  |
|  | 6. Juchipila River at the northwest of | CPUM-20870 | ON008839 | ON023879 | ON008575 | -- |
|  | Alameda Juarez town; Juchipila | CPUM-20872 | ON008840 | ON023880 | ON008576 | ON008705 |
|  | basin, Zacatecas, Mex. |  |  |  |  |  |
|  |  |  |  |  |  |  |
|  | 7. Juchipila River at Tabachines II | CPUM-5745 | ON008841 | ON023881 | ON008577 | ON008706 |
|  | bridge, Tabasco Town; Juchipila | CPUM-5746 | ON008842 | -- | -- | -- |
|  | basin, Zacatecas, Mex. | CPUM-5747 | ON008843 | ON023882 | ON008578 | ON008707 |
|  |  | CPUM-5748 | ON008844 | ON023883 | ON008579 | ON008708 |
|  |  |  |  |  |  |  |
| *Ictalurus pricei* | 8.- Yaqui River | TNHC30450 | EF491727 | -- | -- | -- |
|  |  |  |  |  |  |  |
|  | 9.- Haupoca River, Papigochic River, Madera, Chihuahua, Mex. | TNHC-21704-10 | NC_029158 | -- | -- | -- |
|  |  |  |  |  |  |  |
|  | 10. Bavispe River at Hondables, | CPUM-7963 | ON008845 | ON023884 | ON008580 | -- |
|  | 8.85 km at east of Colonia |  |  |  |  |  |
|  | Morelos, Agua Prieta town; Yaqui |  |  |  |  |  |
|  | basin, Sonora, Mex. |  |  |  |  |  |
|  |  |  |  |  |  |  |
|  | 11. Bavispe River, 7.2 km northeast | CPUM-8002 | ON008846 | ON023885 | ON008581 | ON008709 |
|  | of Huachinera town; Yaqui basin, | CPUM-8003 | ON008847 | ON023886 | ON008582 | -- |
|  | Chihuahua, Mex. |  |  |  |  |  |
|  |  |  |  |  |  |  |
|  | 12. Bavispe River, east of San. | CPUM-8011 | ON008848 | ON023887 | ON008583 | ON008710 |
|  | Miguelito town; Yaqui basin, |  |  |  |  |  |
|  | Chihuahua, Mex |  |  |  |  |  |
|  |  |  |  |  |  |  |
|  | 13. Guerachi River, 27 km | CPUM-7058 | ON008849 | ON023888 | ON008584 | ON008711 |
|  | southwest of Guachochi; Fuerte | CPUM-7059 | ON008850 | ON023889 | ON008585 | ON008712 |
|  | basin, Chihuahua, Mex. | CPUM-7060 | ON008851 | ON023890 | ON008586 | ON008713 |
|  |  | CPUM-7064 | ON008852 | ON023891 | ON008587 | ON008714 |
|  |  | CPUM-7073 | ON008853 | ON023892 | ON008588 | ON008715 |
|  |  | CPUM-7124 | ON008854 | ON023893 | ON008589 | ON008716 |
|  |  |  |  |  |  |  |
|  | 14. Batopilas River at Batopilas, | -- | EF590206 | -- | -- | -- |
|  | Fuerte Basin, Chihuahua, Mex. | USON-01050-2 | -- | KF536995 | -- | -- |
|  |  | USON-01183-1 | -- | KF536996 | -- | -- |
|  |  | USON-01183-2 | -- | KF536997 | -- | -- |
|  |  | TNHC-21704-11 | -- | KF536999 | -- | -- |
|  |  | TNHC-21704-12 | -- | KF537000 | -- | -- |
|  |  | USON-01050-2 | KJ496299 | -- | -- | -- |
|  |  |  |  |  |  |  |
| *Ictalurus* cf. *pricei* | 15. Stream at Rodeo Town; | USON-01115-1 | EF590207 | KJ019216 | -- | -- |
| Culiacan/San Lorenzo | Culiacan basin, Durango, Mex. | USON-01115-2 | -- | KJ019217 | -- | -- |
|  |  |  |  |  |  |  |
|  | 16. San Lorenzo River at Igualamo | USON-01120-1 | EF590208 | KJ019218 | -- | -- |
|  | town; San Lorenzo basin, |  |  |  |  |  |
|  | Durango, Mex. |  |  |  |  |  |
|  |  |  |  |  |  |  |
| *Ictalurus* cf*.* *lupus* | 17. Ramos River at Allende town; | CPUM-2402 | ON008855 | ON023894 | ON008590 | ON008717 |
| Cuatro Cienegas | Bravo basin, Nuevo Leon, Mex. |  |  |  |  |  |
|  |  |  |  |  |  |  |
|  | 18. Charco Prieto pool at Cuatro | CPUM-6374 | ON008856 | ON023895 | ON008591 | ON008718 |
|  | Cienegas valley; endorheic basin, | CPUM-6377 | ON008857 | ON023896 | ON008592 | ON008719 |
|  | Coahuila, Mex. |  |  |  |  |  |
|  |  |  |  |  |  |  |
| *Ictalurus* cf*.* *lupus* Conchos | 19. Porvenir River 11 km | CPUM-7022 | ON008858 | ON023897 | ON008593 | ON008720 |
|  | southeast of Balleza town; Bravo | CPUM-7023 | ON008859 | ON023898 | ON008594 | ON008721 |
|  | basin, Chihuahua, Mex. | CPUM-7024 | ON008860 | ON023899 | ON008595 | ON008722 |
|  |  | CPUM-7025 |  | -- | ON008596 | ON008723 |
|  |  | CPUM-7026 | ON008861 | ON023900 | ON008597 | ON008724 |
|  |  |  |  |  |  |  |
| *Ictalurus* *lupus* | 20. San Felipe Creek, Del Rio Val | TNHC 29350-1 | AY458885 | -- | -- | -- |
|  | Verde Co., Texas, USA. |  |  |  |  |  |
|  |  |  |  |  |  |  |
|  | 21. Devil River, Val Verde | MF23454 | GQ396782 | -- | -- | -- |
|  | County, Texas, USA. | MF23455 | GQ396783 | -- | -- | -- |
|  |  | MF23456 | GQ396784 | -- | -- | -- |
|  |  | MF23457 | GQ396785 | -- | -- | -- |
|  |  | MF23458 | GQ396786 | -- | -- | -- |
|  |  | MF23654 | GQ396794 | -- | -- | -- |
|  |  | MF23655 | GQ396795 | -- | -- | -- |
|  |  | MF23656 | GQ396796 | -- | -- | -- |
|  |  | MF23657 | GQ396797 | -- | -- | -- |
|  |  | NAFF 1285 | -- | JN026910 | -- | -- |
|  |  | NAFF 1284 | -- | JN026911 | -- | -- |
|  |  |  |  |  |  |  |
|  | 22. Frio River near the city of | MF23446 | GQ396774 | -- | -- | -- |
|  | Leakey, Texas, USA. | MF23447 | GQ396775 | -- | -- | -- |
|  |  | MF23448 | GQ396776 | -- | -- | -- |
|  |  | MF23449 | GQ396777 | -- | -- | -- |
|  |  | MF23450 | GQ396778 | -- | -- | -- |
|  |  | MF23451 | GQ396779 | -- | -- | -- |
|  |  | MF23452 | GQ396780 | -- | -- | -- |
|  |  | MF23453 | GQ396781 | -- | -- | -- |
|  |  |  |  |  |  |  |
|  | 23. Independence River, Terrell county, Texas USA. | MF23614 | GQ396787 | -- | -- | -- |
|  |  | MF23616 | GQ396788 | -- | -- | -- |
|  |  | MF23617 | GQ396789 | -- | -- | -- |
|  |  | MF23618 | GQ396790 | -- | -- | -- |
|  |  | MF23637 | GQ396791 | -- | -- | -- |
|  |  |  |  |  |  |  |
|  | 24. Dark Canyon. Eddy Co. New Mexico, USA. | MSB45448 | AY327267 | -- | -- | -- |
|  |  |  |  |  |  |  |
| *Ictalurus* cf. *lupus* Soto | 26. Stream at the north of la Nutria | CPUM-1746 | ON008862 | ON023901 | ON008568 | ON008725 |
| la Marina | town; Soto la Marina basin, | CPUM-1747 | ON008863 | -- | ON008598 | -- |
|  | Tamaulipas, Mex. |  |  |  |  |  |
|  |  |  |  |  |  |  |
|  | 27. Troncones River at Ciudad | RP-137 | ON008864 | ON023902 | ON008599 | ON008726 |
|  | Victoria; Soto la Marina basin, | RP-138 | ON008865 | ON023903 | ON008600 | ON008727 |
|  | Mex. | RP-139 | ON008866 | ON023904 | ON008601 | ON008728 |
|  |  | RP-140 | ON008867 | ON023905 | ON008602 | ON008729 |
|  |  | RP-146 | ON008868 | -- | -- | -- |
|  |  |  |  |  |  |  |
|  | 28. El Chorrito River, road to el Ojo | RP-435 | ON008869 | ON023906 | ON008603 | ON008730 |
|  | de la Virgen Church at Hidalgo | RP-436 | ON008870 | ON023907 | ON008604 | ON008731 |
|  | town, Soto la Marina Basin, | RP-437 | ON008871 | ON023908 | ON008605 | ON008732 |
|  | Tamaulipas, Mex. | RP-438 | ON008872 | ON023909 | ON008606 | ON008733 |
|  |  |  |  |  |  |  |
|  | 29. Stream at Novillo canyon at | RP-524 | ON008873 | ON023910 | ON008607 | ON008734 |
|  | southeast of Ciudad Victoria; Soto |  |  |  |  |  |
|  | la Marina Basin, Tamaulipas, |  |  |  |  |  |
|  | Mex. |  |  |  |  |  |
|  |  |  |  |  |  |  |
| *Ictalurus dugesii* | 30. Lerma River at Pateo town; | CPUM-45605 | ON008874 | -- | ON008608 | -- |
|  | upper Lerma basin, Michoacan, |  |  |  |  |  |
|  | Mex. |  |  |  |  |  |
|  |  |  |  |  |  |  |
|  | 31. Angulo River at Sabino town; | CPUM-6182 | ON008875 | ON023911 | ON008609 | ON008735 |
|  | middle Lerma basin, Michoacan, | CPUM-6224 | ON008876 | ON023912 | ON008610 | ON008736 |
|  | Mex. | CPUM-6225 | ON008877 | ON023913 | ON008611 | ON008737 |
|  |  | CPUM-6226 | ON008878 | ON023914 | ON008612 | ON008738 |
|  |  |  |  |  |  |  |
|  | 32. Angulo River at Botello town; | CPUM-9212 | ON008879 | -- | -- | -- |
|  | middle Lerma basin, Michoacan, | CPUM-9213 | ON008880 | -- | -- | -- |
|  | Mex. |  |  |  |  |  |
|  |  |  |  |  |  |  |
|  | 33. San Juanico Lake at Cotija | CPUM-6295 | ON008881 | ON023915 | ON008613 | ON008739 |
|  | town; endorheic basin, Michoacan, |  |  |  |  |  |
|  | Mex. |  |  |  |  |  |
|  |  |  |  |  |  |  |
|  | 34. Ameca River at San Blasito | CPUM-1813 | ON008882 | ON023916 | ON008614 | ON008740 |
|  | town; Ameca basin, Nayarit, Mex. | CPUM-2245 | ON008883 | ON023917 | ON008615 | ON008741 |
|  |  | CPUM-2246 | ON008884 | -- | -- | -- |
|  |  | CPUM-2247 | ON008885 | -- | -- | -- |
|  |  |  |  |  |  |  |
|  | 35. Stream at Ahuilota pharm, | CPUM-18949 | ON008886 | ON023918 | ON008616 | -- |
|  | Mascota town; Ameca basin, Jal. | CPUM-18950 | ON008887 | ON023919 | ON008617 | -- |
|  | Mex. | CPUM-18951 | ON008888 | ON023920 | ON008618 | -- |
|  |  | CPUM-18952 | ON008889 | -- | -- | -- |
|  |  | CPUM-18953 | ON008890 | -- | -- | -- |
|  |  |  |  |  |  |  |
|  | 36. Ameca River at Amado Nervo; | CPUM-101 | ON008891 | ON023921 | ON008619 | ON008742 |
|  | Ameca Basin, Nayarit, Mex. | CPUM-102 | ON008892 | ON023922 | -- | -- |
|  |  | CPUM-103 | ON008893 | ON023923 | ON008620 | -- |
|  |  | CPUM-104 | ON008894 | -- | -- | -- |
|  |  | CPUM-106 | ON008895 | ON023924 | ON008621 | ON008743 |
|  |  |  |  |  |  |  |
|  | 37. El Limon stream at Atenguillo | CPUM-44580 | ON008896 | ON023925 | ON008622 | -- |
|  | town; Ameca basin, Jalisco, Mex. | CPUM-44582 | ON008897 | ON023926 | ON008623 | -- |
|  |  |  |  |  |  |  |
|  | 38. Spring at Teuchitlan town; | CPUM-42901 | ON008898 | -- | -- | -- |
|  | Ameca basin, Jalisco, Mex. | CPUM-42902 | ON008899 | -- | -- | -- |
|  |  | CPUM-42903 | ON008900 | -- | -- | -- |
|  |  |  |  |  |  |  |
| *Ictalurus* cf. *dugesii* Armeria | 39. Armeria River at Aguacate | CPUM-5174 | ON008901 | ON023928 | ON008624 | ON008744 |
|  | town, El Grullo; Armeria basin, | CPUM-5175 | ON008902 | -- | -- | -- |
|  | Jalisco, Mex. | CPUM-5176 | ON008903 | -- | -- | -- |
|  |  |  |  |  |  |  |
|  | 40. Armeria River at Las | CPUM-18723 | ON008904 | ON023929 | ON008625 | ON008745 |
|  | Ventanas town, El Grullo; Armeria | CPUM-18724 | ON008905 | ON023930 | ON008626 | ON008746 |
|  | basin, Jalisco, Mex. | CPUM-18726 | ON008906 | ON023931 | ON008627 | ON008747 |
|  |  | CPUM-18727 | ON008907 | ON023932 | ON008628 | ON008748 |
|  |  |  |  |  |  |  |
| *Ictalurus ochoterenai* | 41. Chapala Lake at Chapala town; | CPUM-401 | ON008908 | -- | -- | -- |
|  | Lerma-Chapala basin, Jalisco, | CPUM-402 | ON008909 | -- | -- | -- |
|  | Mex. | CPUM-5014 | ON008910 | ON023933 | ON008629 | ON008749 |
|  |  | CPUM-5015 | ON008911 | -- | -- | -- |
|  |  | CPUM-5018 | ON008912 | ON023934 | ON008630 | ON008750 |
|  |  | CPUM-5019 | ON008913 | ON023935 | ON008631 | ON008751 |
|  |  | CPUM-5021 | ON008914 | -- | -- | -- |
|  |  |  |  |  |  |  |
| *Ictalurus* sp. Nazas | 42. Ramos River, 1.4 km north of | CPUM-1796 | ON008915 | ON023936 | ON008632 | ON008752 |
|  | Ramos town; Nazas basin, | CPUM-1800 | ON008916 | ON023937 | ON008633 | ON008753 |
|  | Durango, Mex. | CPUM-1805 | ON008917 | ON023938 | ON008634 | ON008754 |
|  |  |  |  |  |  |  |
|  | 43. El Olote town, Santiago | CPUM-5987 | ON008918 | ON023939 | ON008635 | ON008755 |
|  | Papasquiaro; Nazas basin, | CPUM-6819 | ON008919 | ON023940 | ON008636 | ON008756 |
|  | Durango, Mex. |  |  |  |  |  |
|  |  |  |  |  |  |  |
|  | 44. El Colorado near El Olote | CPUM-2022 | ON008920 | ON023941 | ON008637 | ON008757 |
|  | town, Santiago Papasquiaro; |  |  |  |  |  |
|  | Nazas Basin, Durango, Mex. |  |  |  |  |  |
|  |  |  |  |  |  |  |
| *Ictalurus mexicanus* | 45. Gallinas River at Jabali town; | MNCN-2872 | ON008921 | ON023942 | ON008638 | ON008758 |
|  | Panuco Basin, San Luis Potosi, | MNCN-2873 | ON008922 | ON023943 | ON008639 | ON008759 |
|  | Mex. |  |  |  |  |  |
|  |  |  |  |  |  |  |
| *Ictalurus australis* | 46. Verde River at north of Rio | MNCN-2901 | ON008923 | ON023944 | ON008640 | ON008760 |
|  | Verde town; Panuco Basin, San |  |  |  |  |  |
|  | Luis Potosi, Mex. |  |  |  |  |  |
|  |  |  |  |  |  |  |
|  | 47. Channel 800m | CPUM-4800 | ON008924 | ON023945 | ON008641 | ON008761 |
|  | northwest of Plazuela town; |  |  |  |  |  |
|  | Panuco River, San Luis Potosi, Mex. |  |  |  |  |  |
|  |  |  |  |  |  |  |
|  | 48. Guayalejo River at Llera town; | RP-469* | ON008925 | ON023946 | ON008642 | ON008762 |
|  |  | mtDNA = *I. punctatus,* nDNA = *I. australis* |  |  |  |  |
|  | Panuco Basin, Tamaulipas, Mex. | RP-470* | ON008926 | ON023947 | ON008643 | ON008763 |
|  |  | mtDNA = *I. punctatus,* nDNA = *I. australis* |  |  |  |  |
|  |  |  |  |  |  |  |
| *Ictalurus punctatus* | 49. Mud River, Logan Co. KY, | INHS 93904 | AY184254 | -- | -- | -- |
|  | USA. |  |  |  |  |  |
|  |  |  |  |  |  |  |
|  | 50. Grande River at Brewster | MF23638 | GQ396792 | -- | -- | -- |
|  | County, Texas, USA. | MF23653 | GQ396793 |  |  |  |
|  |  | MF21957 | GQ396769 | -- | -- | -- |
|  |  |  |  |  |  |  |
|  | 51. Pedernales Guillespie County, Texas, USA. | MF23445 | GQ396773 | -- | -- | -- |
|  |  | MF23444 | GQ396772 | -- | -- | -- |
|  |  |  |  |  |  |  |
|  | 52. Dark canyon, Eddy County, | MSB45448 | AY327267 | -- | -- | -- |
|  | New Mexico, USA. |  |  |  |  |  |
|  |  |  |  |  |  |  |
|  | 53. Brazos River at Walter | MF22219 | GQ396770 | -- | -- | -- |
|  | County, Texas, USA. | MF22222 | GQ396771 | -- | -- | -- |
|  |  |  |  |  |  |  |
|  | 54. LaMoine River at Highway 61 | INHS 47559 | AY184253 | -- | -- | -- |
|  | bridge, McDonough County. |  |  |  |  |  |
|  | Illinois, USA. |  |  |  |  |  |
|  |  |  |  |  |  |  |
|  | 55. Concho River, Concho | -- | GQ396767 | -- | -- | -- |
|  | County, Texas USA. |  |  |  |  |  |
|  |  |  |  |  |  |  |
|  | 56. Sabinas River 9.8 km | CPUM-2298 | ON008927 | -- | -- | -- |
|  | northwest of Ciudad Muzquiz; Bravo basin, Coahuila, Mex. | CPUM-2299* | ON008928 | ON023948 | ON008644 | ON008764 |
|  |  | mtDNA = *I. punctatus* |  |  |  |  |
|  |  | nDNA = *I. lupus* |  |  |  |  |
|  |  | CPUM-2300 | ON008929 | ON023949 | ON008645 | -- |
|  |  |  |  |  |  |  |
|  | 57. Falcon Dam 1.4 km north of. | CPUM-1699 | ON008930 | -- | -- | -- |
|  | Nueva Ciudad Guerrero; Bravo | CPUM-1707 | ON008931 | ON023950 | ON008646 | ON008765 |
|  | basin, Tamaulipas, Mex. |  |  |  |  |  |
|  |  |  |  |  |  |  |
|  | 58. Salado River 2.5 km southeast | CPUM-1711 | ON008932 | ON023951 | ON008647 | -- |
|  | of Rancho La Peñita; Bravo River, |  |  |  |  |  |
|  | Coahuila, Mex. |  |  |  |  |  |
|  |  |  |  |  |  |  |
|  | 59. Salado River de los nadadores, | MXIII540 | -- | GU806974 | -- | -- |
|  | Grande River Basin, Progreso | MXIII542 | -- | GU806972 | -- | -- |
|  | Town, Coahuila, Mex. | MXIII541 | -- | GU806971 | -- | -- |
|  |  |  |  |  |  |  |
|  |  |  |  |  |  |  |
|  | 60. Vicente Guerrero Dam; Soto la | CPUM-1692 | ON008933 | ON023952 | ON008648 | ON008766 |
|  | Marina Basin, Tamaulipas, Mex. |  |  |  |  |  |
|  |  |  |  |  |  |  |
|  | 61. Oyul Dam 2 km southeast of | CPUM-1714 | ON008934 | -- | -- | -- |
|  | Antiguo Morelos town; Panuco | CPUM-1715 | ON008935 | ON023953 | ON008649 | ON008767 |
|  | River, Tamaulipas, Mex |  |  |  |  |  |
|  |  |  |  |  |  |  |
|  | 62. Tampaon River south of | CPUM-1717 | ON008936 | ON023954 | ON008650 | ON008768 |
|  | Tamuin town; Panuco basin, San | CPUM-1725 | ON008937 | -- | -- | -- |
|  | Luis Potosi, Mex. |  |  |  |  |  |
|  |  |  |  |  |  |  |
|  | 63. Tamasopo River at Agua | CPUM-1826 | ON008938 | -- | -- | -- |
|  | Buena town; Panuco basin, San |  |  |  |  |  |
|  | Luis Potosi, Mex. |  |  |  |  |  |
|  |  |  |  |  |  |  |
|  | 64. Amajac River 245 m south of | CPUM-3656 | ON008939 | -- | -- | -- |
|  | Zacatipan town; Panuco basin, San |  |  |  |  |  |
|  | Luis Potosi, Mex. |  |  |  |  |  |
|  |  |  |  |  |  |  |
|  | 65. San Pedro River east of | CPUM-3643 | ON008940 | -- | -- | -- |
|  | Orizatlan town; Panuco basin, San |  |  |  |  |  |
|  | Luis Potosi, Mex. |  |  |  |  |  |
|  |  |  |  |  |  |  |
|  | 66. Santa Maria River at Salitrillo | MNCN-2902* | ON008941 | ON023955 | ON008651 | ON008769 |
|  |  | mtDNA = *I. punctatus* |  |  |  |  |
|  |  | nDNA = *I. mexicanus* |  |  |  |  |
|  | town; Panuco basin, San Luis |  |  |  |  |  |
|  | Potosi, Mex. |  |  |  |  |  |
|  |  |  |  |  |  |  |
|  | 67. Pantepec River 480 m | CPUM-3236 | ON008942 | ON023956 | ON008652 | ON008770 |
|  | northeast of Nuevo Carrizal town, | CPUM-3237 | ON008943 | ON023957 | ON008653 | ON008771 |
|  | road Pantepec-Mecalapa; Tuxpan | CPUM-3475 | ON008944 | -- | ON008654 | -- |
|  | basin,Veracruz, Mex. | CPUM-3474* | ON008945 | ON023958 | ON008654 | ON008772 |
|  |  | mtDNA = *I. punctatus* |  |  |  |  |
|  |  | nDNA = *I. mexicanus* |  |  |  |  |
|  |  |  |  |  |  |  |
|  |  |  |  |  |  |  |
|  | 68. Vinazco River north of | CPUM-5507 | ON008946 | -- | -- | -- |
|  | Aguacate de Vinazco town; |  |  |  |  |  |
|  | Tuxpan basin,Veracruz, Mex. |  |  |  |  |  |
|  |  |  |  |  |  |  |
|  | 69. Moctezuma River south of | CPUM-7956 | ON008947 | ON023959 | ON008655 | ON008773 |
|  | Caserio de Terapa town; Yaqui | CPUM-7957 | ON008948 | ON023960 | ON008656 | ON008774 |
|  | basin, Chihuahua, Mex. |  |  |  |  |  |
|  |  |  |  |  |  |  |
|  |  |  |  |  |  |  |
|  | 70. Nazas River in Peñasco at San | CPUM-6063 | ON008949 | ON023961 | ON008657 | ON008775 |
|  | Rafael de Jicorica; Nazas basin, | CPUM-6064 | ON008950 | ON023962 | ON008658 | ON008776 |
|  | Durango, Mex. | CPUM-6083 | ON008951 | ON023963 | ON008659 | ON008777 |
|  |  |  |  |  |  |  |
|  | 71. Nazas River at bridge 5.3 km | CPUM-6176 | ON008952 | ON023964 | ON008660 | ON008778 |
|  | west of Nazas town; Nazas basin, |  |  |  |  |  |
|  | Durango, Mex. |  |  |  |  |  |
|  |  |  |  |  |  |  |
|  | 72. Ramos River south of the | CPUM-5985 | ON008953 | ON023965 | ON008661 | ON008779 |
|  | Olote town; Nazas basin, Durango, |  |  |  |  |  |
|  | Mex. |  |  |  |  |  |
|  |  |  |  |  |  |  |
|  | 73. Ramos River Road to the Olote | CPUM-2021 | ON008954 | -- | -- | -- |
|  | town to Colorado; Nazas basin, | CPUM-2023 | ON008955 | ON023966 | ON008662 | ON008780 |
|  | Durango, Mex. |  |  |  |  |  |
|  |  |  |  |  |  |  |
|  | 74. San Pedro Lagunillas dam; | CPUM-108 | ON008956 | -- | ON008663 | ON008781 |
|  | Huicicila-San Blas basin, Nayarit, | CPUM-109 | ON008957 | ON023967 | ON008664 | ON008782 |
|  | Mex. | CPUM-110 | ON008958 | ON023968 | ON008665 | ON008783 |
|  |  | CPUM-111 | ON008959 | ON023969 | ON008666 | -- |
|  |  | CPUM-112 | ON008960 | ON023970 | ON008667 | ON008784 |
|  |  |  |  |  |  |  |
|  | 75. Animas River 2.5 km | CPUM-1749 | ON008961 | -- | -- | ON008785 |
|  | southwest of Cebadilla town; |  |  |  |  |  |
|  | Balsas basin, Michoacan, Mex. |  |  |  |  |  |
|  |  |  |  |  |  |  |
|  | 76. Balsas River 7 km south of | CPUM-1775 | ON008962 | -- | -- | ON008786 |
|  | Ciudad Altamirano town; Balsas |  |  |  |  |  |
|  | basin, Guerrero, Mex. |  |  |  |  |  |
|  |  |  |  |  |  |  |
|  | 77. El Tesorero dam; Santiago | CPUM-5724 | ON008963 | ON023971 | ON008668 | -- |
|  | basin, Zacatecas, Mex. |  |  |  |  |  |
|  |  |  |  |  |  |  |
|  | 78. Tetiteco River, Road Amado | CPUM-103 | ON008964 | -- | -- | ON008787 |
|  | Nervo to Valle Verde; Ameca |  |  |  |  |  |
|  | basin Nayarit, Mex. |  |  |  |  |  |
|  |  |  |  |  |  |  |
|  | 79. Fleuve St-Laurent, riviere St- | ROM:ICH:BCF-0114-2 | -- | EU524677 | -- | -- |
|  | Nicolas, Quebec, Canada. | ROM:ICH:BCF-0114-1 | -- | EU524678 | -- | -- |
|  |  | NXG2013454 | -- | KX145159 | -- | -- |
|  |  |  |  |  |  |  |
|  | 80. Thames River, Ontario, | ROM:ICH:BCF-0501-2 | EU524679 | -- | -- | -- |
|  | Canada. | ROM:ICH:BCF-0501-1 | EU524680 | -- | -- | -- |
|  |  | ROM:ICH:BCF-0394-1 | EU524683 | -- | -- | -- |
|  |  |  |  |  |  |  |
|  | 81. Fleuve St-Laurent, riviere | BIOUG<CAN>:BCF-0115-2 | EU524685 | -- | -- | -- |
|  | Richelieu, Quebec, Canada | BIOUG<CAN>:BCF-0115-1 | EU524686 | -- | -- | -- |
|  |  |  |  |  |  |  |
|  |  |  |  |  |  |  |
|  |  |  |  |  |  |  |
|  |  |  |  |  |  |  |
|  | 82. Baie Mississippi, Quebec, | BIOUG<CAN>:BCF-0113-1 | EU524106 | -- | -- | -- |
|  | Canada. |  |  |  |  |  |
|  |  |  |  |  |  |  |
|  | 83. Lemke Lake, Ontario, Canada. | NXG2012138 | -- | KX145470 | -- | -- |
|  |  | NXG201386 | -- | KX145476 | -- | -- |
|  |  |  |  |  |  |  |
|  | 84.Thames River, Ontario, Canada. | NXG201378 | -- | KX145334 | -- | -- |
|  |  | ROM:ICH:BCF-0394-1 | -- | EU524683 | -- | -- |
|  |  |  |  |  |  |  |
|  | 85. Grand River, Ontario, Canada. | NXG201210 | -- | KX145299 | -- | -- |
|  |  |  |  |  |  |  |
|  | 86. Pennsylvania, USA. | ANSP:FISH:180368 | EU490865 | -- | -- | DQ492511 |
|  |  |  |  |  |  |  |
|  | 87. Sipsey River, Tuscaloosa, | NAFF 4118 | JN026912 | -- | -- | -- |
|  | Alabama, USA. |  |  |  |  |  |
|  |  |  |  |  |  |  |
|  | 88. Michigan Lake | NEFC_F16-262 | MF621722 | -- | -- | -- |
|  |  | NEFC_F16-568 | MF621721 | -- | -- | -- |
|  |  | NEFC_F16-259 | MF621720 | -- | -- | -- |
|  |  | NEFC_F16-049 | MF621718 | -- | -- | -- |
|  |  | NEFC_F16-045 | MF621717 | -- | -- | -- |
|  |  | NEFC_F16-263 | MF621716 | -- | -- | -- |
|  |  |  |  |  |  |  |
| *Ictalurus* *furcatus* | 89. Mississippi River, Ramsey | -- | KM264126 | -- | -- | -- |
|  | Co., Mississippi basin, MO, USA. |  |  |  |  |  |
|  |  |  |  |  |  |  |
|  | 90. Tamasopo River at Agua | CPUM-1873 | ON008965 | ON023972 | ON008669 | ON008788 |
|  | Buena town; Panuco basin, San |  |  |  |  |  |
|  | Luis Potosi, Mex. |  |  |  |  |  |
|  |  |  |  |  |  |  |
|  | 91. Tampaon River south of | MNCN-2963 | ON008966 | ON023973 | ON008670 | -- |
|  | Tamuin town; Panuco basin, San |  |  |  |  |  |
|  | Luis Potosi, Mex. |  |  |  |  |  |
|  |  |  |  |  |  |  |
|  | 92. Papaloapan River at | CPUM-3226 | ON008967 | ON023974 | ON008671 | ON008789 |
|  | Tlacotalpan; Papaloapan basin, | CPUM-3227 | -- | ON023975 | ON008672 | -- |
|  | Veracruz, Mex. | CPUM-3228 | ON008968 | ON023976 | ON008673 | ON008790 |
|  |  | CPUM-3229 | -- | ON023977 | ON008674 | -- |
|  |  |  |  |  |  |  |
|  | 93. Grande River | -- | ON022107 | -- | -- | -- |
|  |  |  |  |  |  |  |
|  |  |  |  |  |  |  |
|  | 94. Corte at Coatzacoalcos River, | CPUM-264 | ON008969 | -- | -- | ON008791 |
|  | west of Cuauhtémoc; Coatzacoalcos |  |  |  |  |  |
|  | basin, Veracruz, Mex. |  |  |  |  |  |
|  |  |  |  |  |  |  |
|  | 95. Conchos River northeast of | CPUM-2030 | ON008970 | ON023978 | ON008675 | ON008792 |
|  | Zaragoza town; Conchos basin, |  |  |  |  |  |
|  | Chihuahua, Mex. |  |  |  |  |  |
|  |  |  |  |  |  |  |
|  | 96. Louisiana, Cameron Parish, | KUT 1736 | -- | KF929995 | -- | -- |
|  | Superior Canal, Calcasieu Lake. |  |  |  |  |  |
|  |  |  |  |  |  |  |
| *Ictalurus* *meridionalis* | 97. Lagoon at Crooked Tree | CPUM-4567 | ON008971 | ON023979 | ON008676 | ON008793 |
|  | causeway, Crooked Tree, Rio |  |  |  |  |  |
|  | Hondo Basin, Belize. |  |  |  |  |  |
|  |  |  |  |  |  |  |
|  | 98. Peten, Guatemala. | MX657 | -- | EU751844 | -- | -- |
|  |  |  |  |  |  |  |
| *Ictalurus* *balsanus* | 99. Amacuzac River at Huixastla; | UAEM-1IB | ON008972 | ON023980 | ON008677 | ON008794 |
|  | Balsas basin, Morelos, Mex. | UAEM-2IB | ON008973 | ON023981 | ON008678 | ON008795 |
|  |  | UAEM-3IB | ON008974 | ON023982 | ON008679 | ON008796 |
|  |  | UAEM-4IB | ON008975 | ON023983 | ON008680 | ON008797 |
|  |  | CPUM-3251 | ON008976 | -- | -- | -- |
|  |  | CPUM-3259 | ON008977 | -- | -- | -- |
|  |  |  |  |  |  |  |
|  | 100. Mixteco River west of | MNCN-1912 | ON008978 | ON023984 | ON008681 | -- |
|  | Tecomatlan town; Balsas basin, | MNCN-1915 | -- | ON023985 | ON008682 | -- |
|  | Morelos, Mex. |  |  |  |  |  |
|  |  |  |  |  |  |  |
|  | 101. Poliutla River at San José | MNCN-2031 | ON008979 | ON023986 | -- | -- |
|  | Poliutla town; Balsas basin, |  |  |  |  |  |
|  | Guerrero, Mex. |  |  |  |  |  |
|  |  |  |  |  |  |  |
|  | 102. Placeres de Oro River at | MNCN-2032 | ON008980 | -- | -- | -- |
|  | Zihuaquio bridge; Balsas basin, | MNCN-2038 | ON008981 | -- | -- | -- |
|  | Guerrero, Mex. |  |  |  |  |  |
|  |  |  |  |  |  |  |
|  | 103. Mixteco River at Coatlan del Rio. | MNCN-1893 | ON008982 | -- | -- | -- |
|  | town; Balsas basin, Morelos, Mex |  |  |  |  |  |
|  |  |  |  |  |  |  |
|  | 104. Tlapaneca River at Tlapa town; | MNCN-1957 | ON008983 | -- | -- | ON008798 |
|  | Balsas basin, Guerrero, Mex. |  |  |  |  |  |
|  |  |  |  |  |  |  |
|  | 105. Zitacuaro River at Tuzantla | MNCN-3092 | -- | -- | -- | ON008799 |
|  | town; Balsas basin, Michoacan, | MNCN-3094 | ON008984 | -- | -- | -- |
|  | Mex. | MNCN-1309 | ON008985 |  |  |  |
|  |  |  |  |  |  |  |
|  | 106. Tuxpan River at Jungapeo | CPUM-45461 | ON008986 | -- | -- | -- |
|  | Town; Balsas basin, Michoacan, | CPUM-45464 | ON008987 | ON023987 | ON008683 | ON008800 |
|  | Mex. | CPUM-45466 | ON008988 | ON023988 | ON008684 | ON008801 |
|  |  | CPUM-45467 | ON008989 | ON023989 | ON008685 | ON008802 |
|  |  |  | ON008990 | -- | ON008686 | -- |
|  | 107. El Marquez River at south of | CPUM-4994 | ON008991 | ON023990 | ON008687 | ON008803 |
|  | Chauz town; Balsas basin, |  |  |  |  |  |
|  | Michoacan, Mex. |  |  |  |  |  |
|  |  |  |  |  |  |  |
|  | 108. Las Trojes River at Las Trojes | CPUM-1752 | ON008991 | ON023991 | ON008687 | ON008804 |
|  | town; Balsas basin, Michoacan, |  |  |  |  |  |
|  | Mex. |  |  |  |  |  |
|  |  |  |  |  |  |  |
|  | 109. Huetamo River at La Florida | CPUM-1762 | ON008992 | -- | -- | -- |
|  | bridge; Balsas basin, Michoacan, | CPUM-1773 | ON008993 | -- | -- | -- |
|  | Mex. |  |  |  |  |  |
|  |  |  |  |  |  |  |
| *Prietella phreatophila* | 110. Sotano de Amezcua at Ciudad | TNHC 24986 | AY458884 | -- | -- | -- |
|  | Acuña; Bravo basin, Coahuila, |  |  |  |  |  |
|  | Mex. |  |  |  |  |  |
|  |  |  |  |  |  |  |
| *Pylodichthys olivaris* | 111. Porvenir River at 3.2 km | CPUM-7031 | ON008994 | ON023992 | ON008688 | ON008805 |
|  | southeast of Balleza town; Bravo |  |  |  |  |  |
|  | basin, Chihuahua, Mex. |  |  |  |  |  |
|  |  |  |  |  |  |  |
|  | 112. Guayalejo River, Llera town; | RP-468 | ON008995 | ON023993 | ON008689 | ON008806 |
|  | Panuco basin, Tamaulipas, Mex. |  |  |  |  |  |
|  |  |  |  |  |  |  |
|  | 113. Laboratorio de Genética para la Conservación, Centro de Investigaciones Biológicas del Noroeste, La Paz, Baja California Sur, México, | LGC_ADN2186 | ON008996 | ON023994 | ON008690 | ON008807 |
|  |  | (PyOR970720-5) |  |  |  |  |
|  |  |  |  |  |  |  |
| *Noturus gyrinus* | 114. Cummins Creek, Colorado Co, | TNHC 25004 | AY458890 | -- | -- | -- |
|  | Texas, USA. |  |  |  |  |  |
|  |  |  |  |  |  |  |
|  | 115. Laboratorio de Genética para la Conservación, Centro de Investigaciones Biológicas del Noroeste, La Paz, Baja California Sur, México, | LGC_ADN2182 (Noturus89704XX-3) | ON008997 | -- | -- | -- |
|  |  |  |  |  |  |  |
| *Noturus flavus* | 116. Laboratorio de Genética para la Conservación, Centro de Investigaciones Biológicas del Noroeste, La Paz, Baja California Sur, México, | LGC_ADN 2177(NFw470530-2) | ON008998 | ON023995 | ON008691 | ON008808 |
|  |  |  |  |  |  |  |
| *Noturus insignis* | 117. Laboratorio de Genética para la Conservación, Centro de Investigaciones Biológicas del Noroeste, La Paz, Baja California Sur, México, | LGC_ADN2180 (NINC970526-2) | ON008999 | -- | ON008692 | ON008809 |
|  |  |  |  |  |  |  |
| *Ameiurus natalis* | 118. Pin Oak Creek, Bastrop Co., | TNHC 25769 | AY458888 | -- | -- | -- |
|  | Texas, USA. |  |  |  |  |  |
|  |  |  |  |  |  |  |
|  | 119. Laboratorio de Genética para la Conservación, Centro de Investigaciones Biológicas del Noroeste, La Paz, Baja California Sur, México, | LGC_ADN2174(AnBA9706176) | ON009000 | ON023996 | ON008693 | ON008810 |
|  |  |  |  |  |  |  |
| *Cranoglanis bouderius* | 120. Hongshui River in Guangxi | -- | NC_008280 | NC_008280 | NC_008280 | DQ492514 |
|  | province, China |  |  |  |  |  |
|  |  |  |  |  |  |  |
| *Arius arius* | 121. El Corte Coatzacoalcos, | 263 | ON009002 | -- | -- | -- |
|  | Coatzacoalcos Basin, Veracruz, Mex. |  |  |  |  |  |
|  |  |  |  |  |  |  |
| *Pangasianodon* | 122. Guangzhou, China | -- | NC_021752 | NC_021752 | NC_021752 | KR080265 |
| *hypophthalmus* |  |  |  |  |  |  |
|  |  |  |  |  |  |  |
| *Rita rita* | 123. Southern Asia | DQ119457 | -- | -- | -- | -- |

**Supporting information**

**Appendix S2. Time calibrated analysis procedure performed in BEAST**

A time-calibrated tree was reconstructed for 13 individuals, including all described species and all populations that show a genetic divergence ≥ 1.5% in the *cytb* gene (except *Ictalurus lupus* and *I.*cf. *pricei* Culiacan/San Lorenzo due to the lack of sequences for the *atpase8/6* and *RAG1*), plus *Noturus flavus*, *Pylodictis olivaris,* and *Ameirus natalis* as the outgroup using BEAST v.1.8.4 (Drummond et al., 2012). We use the best fit models of nucleotide substitution estimated for each locus (*cytb*: GTR+G, *cox1:* HKY+I+G, *atpase8/6:* GTR+G, *RAG1:* HKY), implement a relaxed clock with an uncorrelated lognormal model, and a Yule speciation process. Four fossil constraints were used as calibration points; the fossil of †*Ictalurus rhaeas* dated at Eocene-Oligocene (37-30 Ma) (Prothero, 1995) is regarded as the oldest fossil of the genus *Ictalurus*, accordingly was used to constrain the crown group of the genus to a hard minimum bound of 30 Ma; in addition, and based on the information that the stem group of *Ictalurus* was located at ̴ 40 Ma (*sensu* Arce-H et al., 2016), a soft upper bound of 40 Ma was included using a lognormal prior. The fossil †*Ictalurus lambda* Hubbs and Hibbard, 1951 dated at the middle-late Miocene (10.3-13.6 Ma, Arce-H et al., 2016) that shares many features with *I. furcatus*, but, as well present exclusive diagnostic features (Lundberg, 1975), was used as a calibration point for the crown clade of *furcatus* group, setting a hard minimum bound of 10 Ma. For the third calibration point, we use the record fossil of the extant species *Ictalurus punctatus* dated at early-middle Miocene (15.9-18.9 Ma, Arce-H et al., 2016) to constrain the most recent common ancestor of the *I. punctatus* to a hard minimum bound of 16 Ma. For the last calibration point (C4), the species fossil †*Ictalurus spodius* Smith, 1987 found in Chapala Lake (Smith, 1987), dated at the Late Pleistocene (1.0-1.5 Ma, Arce-H et al., 2016) and highly related with the extant species *I. dugesii* (Smith, 1987), was used to constrain the most recent common ancestor of this species (C4) to a hard minimum bound of 1 Ma with a soft upper bound of 11 Ma based on the formation of the ancient Chapala Lake (Late Miocene, Israde-Alcántara, 1999). Since the lack of appropriate information to determine the shape of the lognormal distribution (Ho and Phillips, 2009), for the case of the latter three calibration points, C2, C3, and C4, a soft upper bound of 40 Ma was established using an exponential prior. We conducted three independent analyses of 300 million generations, sampling every 3000 generations. Convergence and stationary distribution of the runs were verified by the effective sample size for all parameters (ESS >200) as visualized in Tracer 1.5. Ten percent of the generations were discarded as burn-in and pooled using the module Log-Combiner in the BEAST package. The maximum clade credibility species tree was obtained by the module Tree-Annotator in the BEAST package.

**References**

Arce-H M., J. G. Lundberg and M. A. O’Learya. 2016. Phylogeny of the North American catfish family Ictaluridae Teleostei: Siluriformes) combining morphology, genes and fossils. Cladistics 1-23.

Drummond A.J., Suchard M.A., Xie D., Rambaut A. 2012. Bayesian phylogenetics with BEAUti and the BEAST 1.7. Molecular Biology and Evolution, 29, 1969-1973.

Ho S.Y.W.,and Phillips M.J. 2009. Accounting for calibration uncertainty in phylogenetic estimation of evolutionary divergence times. Systematic Biology, 58:367-380.

Israde-Alcántara I. 1999. Los lagos volcánicos y tectónicos de Michoacán. En Garduño-Monroy V.H., Corona-Chávez P., Israde-Alcántara I., Menella,L. Arreyque E., Bigioggero B., Chiesa S. eds. *Carta Geológica de Michoacán*. Universidad Michoacana de San Nicolas de Hidalgo. 45-73.

Lundberg, J.G., 1975. The fossil catfishes of North America. University of Michigan Papers on Paleontology 11: 61.

Prothero, D.R., 1995. Geochronology and magnetostratigraphy of Paleogene North American land mammal ‘ages’: an update. Society of Economic Paleontolgists and Minerallogists. Special Publication, 54, 305–315.

Smith M.L., 1987. Osteology and systematics of the fossil catfishes (genus *Ictalurus*) of Central Mexico. Journal of Paleontology 61: 380–387.

**Table S2**. Indexes and other parameters from substitution saturation tests for codon 1, 2 and 3 using Xia’s method (Xia et al., 2003; Xia & Lemey, 2009) in DAMBE 6.4.29 (Xia, 2013). NumOTU= number of operational units; Iss= index of substitutional saturation; Iss.cSym= critical index of substitutional saturation assuming a symmetrical topology; Iss.cAsym= critical index of substitutional saturation assuming an asymmetrical topology.

| Codon position | NumOTU | Iss | Iss.cSym | T | DF | P | Iss.cAsym | T | DF | P |
| --- | --- | --- | --- | --- | --- | --- | --- | --- | --- | --- |
| *cytb* 1 pos | 4 | 0.275 | 0.785 | 22.368 | 363 | 0.000 | 0.756 | 21.069 | 363 | 0.000 |
|  | 8 | 0.261 | 0.739 | 21.255 | 363 | 0.000 | 0.628 | 16.338 | 363 | 0.000 |
|  | 16 | 0.264 | 0.694 | 19.636 | 363 | 0.000 | 0.486 | 10.102 | 363 | 0.000 |
|  | 32 | 0.264 | 0.687 | 19.928 | 363 | 0.000 | 0.358 | 4.401 | 363 | 0.000 |
| *cytb* 2 pos | 4 | 0.062 | 0.785 | 64.075 | 363 | 0.000 | 0.756 | 61.450 | 363 | 0.000 |
|  | 8 | 0.055 | 0.739 | 59.979 | 363 | 0.000 | 0.628 | 50.290 | 363 | 0.000 |
|  | 16 | 0.052 | 0.694 | 57.847 | 363 | 0.000 | 0.486 | 39.054 | 363 | 0.000 |
|  | 32 | 0.053 | 0.687 | 56.146 | 363 | 0.000 | 0.358 | 26.958 | 363 | 0.000 |
| *cytb* 3 pos | 4 | 0.062 | 0.785 | 64.075 | 363 | 0.000 | 0.756 | 61.450 | 363 | 0.000 |
|  | 8 | 0.055 | 0.739 | 59.979 | 363 | 0.000 | 0.628 | 50.290 | 363 | 0.000 |
|  | 16 | 0.052 | 0.694 | 57.847 | 363 | 0.000 | 0.486 | 39.054 | 363 | 0.000 |
|  | 32 | 0.053 | 0.687 | 56.146 | 363 | 0.000 | 0.358 | 26.958 | 363 | 0.000 |
| *cox1* 1 pos | 4 | 0.018 | 0.776 | 79.811 | 202 | 0.000 | 0.767 | 78.796 | 202 | 0.000 |
|  | 8 | 0.017 | 0.734 | 74.354 | 202 | 0.000 | 0.636 | 64.220 | 202 | 0.000 |
|  | 16 | 0.017 | 0.644 | 66.171 | 202 | 0.000 | 0.455 | 46.245 | 202 | 0.000 |
|  | 32 | 0.018 | 0.690 | 72.153 | 202 | 0.000 | 0.375 | 38.316 | 202 | 0.000 |
| *cox1* 2 pos | 4 | 0.006 | 0.776 | 221.496 | 202 | 0.000 | 0.767 | 218.725 | 202 | 0.000 |
|  | 8 | 0.005 | 0.734 | 183.521 | 202 | 0.000 | 0.636 | 158.901 | 202 | 0.000 |
|  | 16 | 0.005 | 0.644 | 165.381 | 202 | 0.000 | 0.455 | 116.506 | 202 | 0.000 |
|  | 32 | 0.005 | 0.690 | 179.189 | 202 | 0.000 | 0.375 | 96.669 | 202 | 0.000 |
| *cox1* 3 pos | 4 | 0.214 | 0.776 | 19.777 | 202 | 0.000 | 0.767 | 19.437 | 202 | 0.000 |
|  | 8 | 0.207 | 0.734 | 18.177 | 202 | 0.000 | 0.636 | 14.809 | 202 | 0.000 |
|  | 16 | 0.205 | 0.644 | 15.278 | 202 | 0.000 | 0.455 | 8.698 | 202 | 0.000 |
|  | 32 | 0.208 | 0.690 | 16.991 | 202 | 0.000 | 0.375 | 5.868 | 202 | 0.000 |
| *atpase8/6* 1 pos | 4 | 0.038 | 0.780 | 81.531 | 286 | 0.000 | 0.756 | 78.951 | 286 | 0.000 |
|  | 8 | 0.040 | 0.733 | 74.053 | 286 | 0.000 | 0.626 | 62.590 | 286 | 0.000 |
|  | 16 | 0.043 | 0.674 | 66.823 | 286 | 0.000 | 0.470 | 45.168 | 286 | 0.000 |
|  | 32 | 0.047 | 0.682 | 68.137 | 286 | 0.000 | 0.354 | 32.893 | 286 | 0.000 |
| *atpase8/6* 2 pos | 4 | 0.180 | 0.780 | 28.627 | 286 | 0.000 | 0.756 | 27.508 | 286 | 0.000 |
|  | 8 | 0.184 | 0.733 | 25.482 | 286 | 0.000 | 0.626 | 20.506 | 286 | 0.000 |
|  | 16 | 0.182 | 0.674 | 22.997 | 286 | 0.000 | 0.470 | 13.453 | 286 | 0.000 |
|  | 32 | 0.185 | 0.682 | 23.562 | 286 | 0.000 | 0.354 | 8.013 | 286 | 0.000 |
| *atpase8/6* 3 pos | 4 | 0.086 | 0.780 | 46.451 | 286 | 0.000 | 0.756 | 44.880 | 286 | 0.000 |
|  | 8 | 0.084 | 0.733 | 40.760 | 286 | 0.000 | 0.626 | 34.024 | 286 | 0.000 |
|  | 16 | 0.086 | 0.674 | 36.945 | 286 | 0.000 | 0.470 | 24.101 | 286 | 0.000 |
|  | 32 | 0.091 | 0.682 | 37.086 | 286 | 0.000 | 0.54 | 16.494 | 286 | 0.000 |
| *rag1* 1pos | 4 | 0.024 | 0.784 | 98.486 | 344 | 0.000 | 0.756 | 94.799 | 344 | 0.000 |
|  | 8 | 0.024 | 0.737 | 86.868 | 344 | 0.000 | 0.627 | 73.480 | 344 | 0.000 |
|  | 16 | 0.025 | 0.690 | 80.246 | 344 | 0.000 | 0.482 | 55.112 | 344 | 0.000 |
|  | 32 | 0.026 | 0.686 | 82.742 | 344 | 0.000 | 0.356 | 41.359 | 344 | 0.000 |
| *rag1* 2pos | 4 | 0.014 | 0.784 | 124.595 | 344 | 0.000 | 0.756 | 119.992 | 344 | 0.000 |
|  | 8 | 0.014 | 0.737 | 111.521 | 344 | 0.000 | 0.627 | 94.573 | 344 | 0.000 |
|  | 16 | 0.014 | 0.690 | 104.501 | 344 | 0.000 | 0.482 | 72.345 | 344 | 0.000 |
|  | 32 | 0.015 | 0.686 | 103.774 | 344 | 0.000 | 0.356 | 52.766 | 344 | 0.000 |
| *rag1* 3pos | 4 | 0.007 | 0.784 | 198.206 | 344 | 0.000 | 0.756 | 190.948 | 344 | 0.000 |
|  | 8 | 0.006 | 0.737 | 183.908 | 344 | 0.000 | 0.627 | 156.240 | 344 | 0.000 |
|  | 16 | 0.006 | 0.690 | 180.333 | 344 | 0.000 | 0.482 | 125.424 | 344 | 0.000 |
|  | 32 | 0.007 | 0.686 | 187.717 | 344 | 0.000 | 0.356 | 96.452 | 344 | 0.000 |
|  |  |  |  |  |  |  |  |  |  |  |

**References**

Xia, X. (2013) DAMBE5: a comprehensive software package for data analysis in molecular biology and evolution. *Molecular Biology and Evolution*, **30**, 1720–1728.

Xia, X. & Lemey, P. (2009) Assessing substitution saturation with DAMBE. *The phylogenetic handbook: a practical approach to Phylogenetic analysis and hypothesis testing* (ed. By Lemey, P.), pp. 615–630.

Xia, X., Xie, Z., Salemi, M., Chen, L. & Wang, Y. (2003) An index of substitution saturation and its application. *Molecular Phylogenetics and Evolution*, **26**, 1–7.

**Table S3.** Uncorrected genetic distances based on *cytb* between distinct species and lineages within the genus *Ictalurus*. Mean values ± standard deviation.

|  | *1* | *2* | *3* | *4* | *5* | *6* | *7* | *8* | *9* | *10* | *11* | *12* | *13* | *14* | *15* | *16* | *17* |
| --- | --- | --- | --- | --- | --- | --- | --- | --- | --- | --- | --- | --- | --- | --- | --- | --- | --- |
| *1. I. dugesii* |  |  |  |  |  |  |  |  |  |  |  |  |  |  |  |  |  |
| *2. I. ochoterenai* | 0.002 ± 0.001 |  |  |  |  |  |  |  |  |  |  |  |  |  |  |  |  |
| *3. I.* cf. *dugesii* Armeria | 0.009 ± 0.003 | 0.007 ± 0.003 |  |  |  |  |  |  |  |  |  |  |  |  |  |  |  |
| *4. I. lupus* | 0.022 ± 0.005 | 0.022 ± 0.005 | 0.027 ± 0.005 |  |  |  |  |  |  |  |  |  |  |  |  |  |  |
| *5. I.* cf. *lupus* Cuatro Cienegas | 0.025 ± 0.005 | 0.023 ± 0.005 | 0.029 ± 0.006 | 0.020 ± 0.004 |  |  |  |  |  |  |  |  |  |  |  |  |  |
| *6. I.* cf. *lupus* Conchos | 0.029 ± 0.005 | 0.028 ± 0.005 | 0.033 ± 0.006 | 0.027 ± 0.005 | 0.019 ± 0.004 |  |  |  |  |  |  |  |  |  |  |  |  |
| *7. I. pricei* | 0.024 ± 0.005 | 0.023 ± 0.005 | 0.028 ± 0.005 | 0.030 ± 0.005 | 0.026 ± 0.005 | 0.032 ± 0.005 |  |  |  |  |  |  |  |  |  |  |  |
| *8. I. mexicanus* | 0.048 ± 0.007 | 0.047 ± 0.007 | 0.053 ± 0.007 | 0.056 ± 0.007 | 0.059 ± 0.008 | 0.062 ± 0.008 | 0.056 ± 0.007 |  |  |  |  |  |  |  |  |  |  |
| *9. I. australis* | 0.051 ± 0.007 | 0.049 ± 0.007 | 0.055 ± 0.007 | 0.058 ± 0.008 | 0.061 ± 0.008 | 0.064 ± 0.008 | 0.058 ± 0.007 | 0.005 ± 0.002 |  |  |  |  |  |  |  |  |  |
| *10. I. punctatus* | 0.073 ± 0.009 | 0.072 ± 0.009 | 0.075 ± 0.009 | 0.078 ± 0.009 | 0.082 ± 0.009 | 0.087 ± 0.009 | 0.076 ± 0.008 | 0.075 ± 0.008 | 0.076 ± 0.008 |  |  |  |  |  |  |  |  |
| *11. I. furcatus* | 0.096 ± 0.009 | 0.095 ± 0.010 | 0.099 ± 0.010 | 0.102 ± 0.010 | 0.099 ± 0.009 | 0.105 ± 0.010 | 0.102 ± 0.010 | 0.108 ± 0.010 | 0.109 ± 0.010 | 0.098 ± 0.009 |  |  |  |  |  |  |  |
| *12. I. meridionalis* | 0.098 ± 0.010 | 0.097 ± 0.010 | 0.100 ± 0.010 | 0.103 ± 0.010 | 0.102 ± 0.010 | 0.107 ± 0.010 | 0.102 ± 0.010 | 0.111 ± 0.010 | 0.113 ± 0.010 | 0.100 ± 0.010 | 0.015 ± 0.004 |  |  |  |  |  |  |
| *13. I. balsanus* | 0.110 ± 0.010 | 0.109 ± 0.010 | 0.112 ± 0.010 | 0.116 ± 0.010 | 0.113 ± 0.010 | 0.121 ± 0.010 | 0.109 ± 0.010 | 0.111 ± 0.010 | 0.112 ± 0.010 | 0.106 ± 0.010 | 0.090 ± 0.009 | 0.089 ± 0.009 |  |  |  |  |  |
| *14. I.* cf*. lupus* Soto La Marina | 0.023 ± 0.005 | 0.021 ± 0.005 | 0.026 ± 0.005 | 0.028 ± 0.005 | 0.030 ± 0.006 | 0.034 ± 0.006 | 0.033 ± 0.006 | 0.061 ± 0.008 | 0.063 ± 0.008 | 0.082 ± 0.009 | 0.102 ± 0.010 | 0.101 ± 0.010 | 0.115± 0.010 |  |  |  |  |
| *15. Ictalurus sp.* Mezquital | 0.024 ± 0.005 | 0.022 ± 0.005 | 0.027 ± 0.005 | 0.034 ± 0.006 | 0.030 ± 0.006 | 0.035 ± 0.006 | 0.018 ± 0.004 | 0.058 ± 0.008 | 0.060 ± 0.008 | 0.072 ± 0.008 | 0.092 ± 0.009 | 0.091 ± 0.009 | 0.105± 0.010 | 0.033± 0.006 |  |  |  |
| *16. I. dugesii* Santiago | 0.021 ± 0.005 | 0.020 ± 0.004 | 0.025 ± 0.005 | 0.033 ± 0.006 | 0.029 ± 0.005 | 0.035 ± 0.006 | 0.019 ± 0.004 | 0.058 ± 0.008 | 0.060 ± 0.008 | 0.076 ± 0.009 | 0.104 ± 0.010 | 0.102 ± 0.010 | 0.112± 0.010 | 0.033± 0.006 | 0.018± 0.004 |  |  |
| *17. I.* cf. *pricei* Culiacan/San Lorenzo | 0.021 ± 0.004 | 0.020 ± 0.004 | 0.025 ± 0.005 | 0.032 ± 0.006 | 0.024 ± 0.005 | 0.031 ± 0.005 | 0.017 ± 0.004 | 0.056 ± 0.007 | 0.057 ± 0.007 | 0.075 ± 0.008 | 0.100 ± 0.009 | 0.095 ± 0.009 | 0.104± 0.009 | 0.031± 0.005 | 0.016± 0.004 | 0.019 ± 0.004 |  |
| *18. Ictalurus* sp. Nazas | 0.020 ± 0.004 | 0.017 ± 0.004 | 0.021 ± 0.005 | 0.031 ± 0.006 | 0.032 ± 0.006 | 0.037 ± 0.006 | 0.032 ± 0.006 | 0.051 ± 0.007 | 0.053 ± 0.007 | 0.073 ± 0.008 | 0.099 ± 0.010 | 0.102 ± 0.010 | 0.109± 0.010 | 0.030± 0.006 | 0.031± 0.005 | 0.033 ± 0.006 | 0.031 ± 0.006 |

**Table S4**. Uncorrected genetic distances based on *cox1* between distinct species and lineages within the genus *Ictalurus*. Mean values ± standard deviation.

|  | *1* | *2* | *3* | *4* | *5* | *6* | *7* | *8* | *9* | *10* | *11* | *12* | *13* | *14* | *15* | *16* | *17* |
| --- | --- | --- | --- | --- | --- | --- | --- | --- | --- | --- | --- | --- | --- | --- | --- | --- | --- |
| *1. I. dugesii* |  |  |  |  |  |  |  |  |  |  |  |  |  |  |  |  |  |
| *2. I. ochoterenai* | 0.001 ± 0.001 |  |  |  |  |  |  |  |  |  |  |  |  |  |  |  |  |
| *3. I.* cf. *dugesii* Armeria | 0.004 ± 0.002 | 0.003 ± 0.002 |  |  |  |  |  |  |  |  |  |  |  |  |  |  |  |
| *4. I. pricei* | 0.026 ± 0.006 | 0.026 ± 0.006 | 0.025 ± 0.006 |  |  |  |  |  |  |  |  |  |  |  |  |  |  |
| *5. I.* cf. *lupus* Conchos | 0.022 ± 0.005 | 0.022 ± 0.005 | 0.022 ± 0.005 | 0.023 ± 0.005 |  |  |  |  |  |  |  |  |  |  |  |  |  |
| *6. I.* cf. *lupus* Cuatro Cienegas | 0.027 ± 0.006 | 0.027 ± 0.007 | 0.027 ± 0.007 | 0.032 ± 0.007 | 0.011 ± 0.003 |  |  |  |  |  |  |  |  |  |  |  |  |
| *7. I. australis* | 0.047 ± 0.008 | 0.047 ± 0.008 | 0.043 ± 0.008 | 0.058 ± 0.009 | 0.050 ± 0.008 | 0.051 ± 0.009 |  |  |  |  |  |  |  |  |  |  |  |
| *8. I. mexicanus* | 0.044 ± 0.008 | 0.044 ± 0.008 | 0.041 ± 0.008 | 0.055 ± 0.009 | 0.047 ± 0.008 | 0.051 ± 0.009 | 0.003 ± 0.002 |  |  |  |  |  |  |  |  |  |  |
| *9. I. punctatus* | 0.057 ± 0.009 | 0.057 ± 0.009 | 0.057 ± 0.009 | 0.067 ± 0.010 | 0.051 ± 0.008 | 0.053 ± 0.009 | 0.069 ± 0.010 | 0.067 ± 0.010 |  |  |  |  |  |  |  |  |  |
| *10. I. furcatus* | 0.090 ± 0.012 | 0.089 ± 0.012 | 0.089 ± 0.012 | 0.101 ± 0.012 | 0.094 ± 0.012 | 0.097 ± 0.012 | 0.094 ± 0.012 | 0.091 ± 0.011 | 0.090 ± 0.012 |  |  |  |  |  |  |  |  |
| *11. I. meridionalis* | 0.093 ± 0.012 | 0.092 ± 0.012 | 0.092 ± 0.012 | 0.105 ± 0.012 | 0.099 ± 0.012 | 0.103 ± 0.013 | 0.100 ± 0.012 | 0.097 ± 0.012 | 0.090 ± 0.011 | 0.013 ± 0.004 |  |  |  |  |  |  |  |
| *12. I. balsanus* | 0.108 ± 0.013 | 0.108 ± 0.013 | 0.108 ± 0.013 | 0.109 ± 0.013 | 0.104 ± 0.012 | 0.103 ± 0.013 | 0.107 ± 0.013 | 0.104 ± 0.012 | 0.095 ± 0.012 | 0.087 ± 0.012 | 0.093 ± 0.012 |  |  |  |  |  |  |
| *13. I.* cf*. lupus* Soto La Marina | 0.027 ± 0.006 | 0.027 ± 0.007 | 0.027 ± 0.007 | 0.036 ± 0.007 | 0.018 ± 0.005 | 0.018 ± 0.006 | 0.055 ± 0.009 | 0.053 ± 0.009 | 0.053 ± 0.009 | 0.092 ± 0.012 | 0.099 ± 0.012 | 0.102 ± 0.013 |  |  |  |  |  |
| *14. Ictalurus* sp. Mezquital | 0.023± 0.006 | 0.023 ± 0.006 | 0.023 ± 0.006 | 0.020 ± 0.005 | 0.023 ± 0.006 | 0.029 ± 0.007 | 0.059 ± 0.010 | 0.057 ± 0.010 | 0.061 ± 0.010 | 0.095 ± 0.012 | 0.098 ± 0.012 | 0.106 ± 0.013 | 0.033 ± 0.007 |  |  |  |  |
| *15. I.* cf. *dugesii* Santiago | 0.026 ± 0.006 | 0.026 ± 0.006 | 0.026 ± 0.006 | 0.018 ± 0.005 | 0.021 ± 0.005 | 0.025 ± 0.006 | 0.056 ± 0.009 | 0.053 ± 0.009 | 0.062 ± 0.010 | 0.092 ± 0.012 | 0.099 ± 0.012 | 0.101 ± 0.013 | 0.029 ± 0.007 | 0.016 ± 0.005 |  |  |  |
| *16. I.* cf. *price* Culiacan/San Lorenzo | 0.024 ± 0.006 | 0.024 ± 0.006 | 0.020 ± 0.005 | 0.016 ± 0.004 | 0.022 ± 0.005 | 0.028 ± 0.007 | 0.055 ± 0.009 | 0.053 ± 0.009 | 0.062 ± 0.009 | 0.095 ± 0.012 | 0.098 ± 0.012 | 0.104 ± 0.013 | 0.033 ± 0.007 | 0.018 ± 0.005 | 0.017 ± 0.005 |  |  |
| *17. Ictalurus* sp. Nazas | 0.024 ± 0.006 | 0.024 ± 0.006 | 0.024 ± 0.006 | 0.029 ± 0.007 | 0.017 ± 0.005 | 0.020 ± 0.006 | 0.049 ± 0.009 | 0.046 ± 0.008 | 0.056 ± 0.009 | 0.091 ± 0.012 | 0.095 ± 0.012 | 0.102 ± 0.012 | 0.020 ± 0.006 | 0.026 ± 0.007 | 0.026 ± 0.007 | 0.026 ± 0.006 |  |
| *18. I. lupus* | 0.028 ± 0.007 | 0.028 ± 0.007 | 0.028 ± 0.007 | 0.034 ± 0.007 | 0.012 ± 0.004 | 0.011 ± 0.004 | 0.051 ± 0.009 | 0.049 ± 0.009 | 0.050 ± 0.009 | 0.093 ± 0.012 | 0.100 ± 0.012 | 0.103 ± 0.013 | 0.019 ± 0.006 | 0.030 ± 0.007 | 0.025 ± 0.006 | 0.030 ± 0.007 | 0.021 ± 0.006 |

**Table S5.** Uncorrected genetic distances based on *atpase8/6* between distinct species and lineages within the genus *Ictalurus*. Mean values ± standard deviation.

|  | *1* | *2* | *3* | *4* | *5* | *6* | *7* | *8* | *9* | *10* | *11* | *12* | *13* | *14* | *15* |
| --- | --- | --- | --- | --- | --- | --- | --- | --- | --- | --- | --- | --- | --- | --- | --- |
| *1. I. ochoterenai* |  |  |  |  |  |  |  |  |  |  |  |  |  |  |  |
| *2. I. dugesii* | 0.001 ± 0.001 |  |  |  |  |  |  |  |  |  |  |  |  |  |  |
| *3. I.* cf. *dugesii* Armeria | 0.006 ± 0.002 | 0.005 ± 0.002 |  |  |  |  |  |  |  |  |  |  |  |  |  |
| *4. I. pricei* | 0.023 ± 0.005 | 0.022 ± 0.005 | 0.027 ± 0.005 |  |  |  |  |  |  |  |  |  |  |  |  |
| *5. I.* cf. *lupus* Conchos | 0.024 ± 0.005 | 0.025 ± 0.005 | 0.030 ± 0.006 | 0.035 ± 0.006 |  |  |  |  |  |  |  |  |  |  |  |
| *6. I.* cf. *lupus* Cuatro Cienegas | 0.021 ± 0.005 | 0.021 ± 0.005 | 0.026 ± 0.005 | 0.034 ± 0.006 | 0.011 ± 0.003 |  |  |  |  |  |  |  |  |  |  |
| *7. I. australis* | 0.056 ± 0.008 | 0.055 ± 0.008 | 0.060 ± 0.008 | 0.058 ± 0.008 | 0.059 ± 0.008 | 0.057 ± 0.008 |  |  |  |  |  |  |  |  |  |
| *8. I. mexicanus* | 0.059 ± 0.008 | 0.058 ± 0.008 | 0.061 ± 0.008 | 0.058 ± 0.008 | 0.067 ± 0.009 | 0.063 ± 0.009 | 0.018 ± 0.004 |  |  |  |  |  |  |  |  |
| *9. I. punctatus* | 0.063 ± 0.008 | 0.063 ± 0.008 | 0.069 ± 0.009 | 0.072 ± 0.009 | 0.073 ± 0.009 | 0.069 ± 0.009 | 0.072 ± 0.009 | 0.079 ± 0.009 |  |  |  |  |  |  |  |
| *10. I. furcatus* | 0.092 ± 0.010 | 0.093 ± 0.010 | 0.093 ± 0.010 | 0.096 ± 0.010 | 0.092 ± 0.010 | 0.092 ± 0.010 | 0.104 ± 0.010 | 0.104 ± 0.010 | 0.078 ± 0.009 |  |  |  |  |  |  |
| *11. I. meridionalis* | 0.094 ± 0.010 | 0.094 ± 0.010 | 0.095 ± 0.010 | 0.097 ± 0.010 | 0.093 ± 0.010 | 0.094 ± 0.010 | 0.103 ± 0.010 | 0.105 ± 0.010 | 0.080 ± 0.009 | 0.005 ± 0.002 |  |  |  |  |  |
| *12. I. balsanus* | 0.122 ± 0.011 | 0.122 ± 0.011 | 0.121 ± 0.011 | 0.127 ± 0.011 | 0.127 ± 0.011 | 0.119 ± 0.011 | 0.122 ± 0.011 | 0.122 ± 0.011 | 0.114 ± 0.010 | 0.089 ± 0.009 | 0.090 ± 0.009 |  |  |  |  |
| *13. I.* cf. *lupus* Soto La Marina | 0.032 ± 0.006 | 0.031 ± 0.006 | 0.036 ± 0.006 | 0.039 ± 0.006 | 0.039 ± 0.007 | 0.040 ± 0.007 | 0.065 ± 0.009 | 0.070 ± 0.009 | 0.076 ± 0.009 | 0.096 ± 0.010 | 0.098 ± 0.010 | 0.127 ± 0.011 |  |  |  |
| *14. Ictalurus* sp.  Mezquitalr | 0.020 ± 0.005 | 0.019 ± 0.005 | 0.024 ± 0.005 | 0.021 ± 0.004 | 0.031 ± 0.006 | 0.033 ± 0.006 | 0.059 ± 0.009 | 0.060 ± 0.008 | 0.074 ± 0.009 | 0.099 ± 0.010 | 0.100 ± 0.010 | 0.124 ± 0.011 | 0.039 ± 0.006 |  |  |
| *15. I.* cf. *dugesii* Santiago | 0.020 ± 0.005 | 0.019 ± 0.005 | 0.024 ± 0.005 | 0.014 ± 0.004 | 0.032 ± 0.006 | 0.028 ± 0.006 | 0.054 ± 0.008 | 0.060 ± 0.008 | 0.068 ± 0.009 | 0.093 ± 0.010 | 0.094 ± 0.010 | 0.121 ± 0.011 | 0.037 ± 0.006 | 0.015 ± 0.004 |  |
| *16. Ictalurus* sp. Nazas | 0.021 ± 0.005 | 0.022 ± 0.005 | 0.025 ± 0.005 | 0.029 ± 0.005 | 0.035 ± 0.006 | 0.029 ± 0.006 | 0.060 ± 0.008 | 0.064 ± 0.008 | 0.071 ± 0.009 | 0.098 ± 0.010 | 0.100 ± 0.010 | 0.119 ± 0.011 | 0.041 ± 0.007 | 0.028 ± 0.005 | 0.021± 0.005 |

**Table S6.** Uncorrected genetic distances based on *RAG1* between distinct species and lineages within the genus *Ictalurus*. Mean values ± standard deviation.

|  | *1* | *2* | *3* | *4* | *5* | *6* | *7* | *8* | *9* | *10* | *11* | *12* | *13* | *14* | *15* |
| --- | --- | --- | --- | --- | --- | --- | --- | --- | --- | --- | --- | --- | --- | --- | --- |
| *1. I. dugesii* |  |  |  |  |  |  |  |  |  |  |  |  |  |  |  |
| *2. I. ochoterenai* | 0.002± 0.001 |  |  |  |  |  |  |  |  |  |  |  |  |  |  |
| *3. I.* cf. *dugesii* Armeria | 0.002± 0.001 | 0.0003± 0.0003 |  |  |  |  |  |  |  |  |  |  |  |  |  |
| *4. I. pricei* | 0.002± 0.001 | 0.0004± 0.0003 | 0.0001± 0.0001 |  |  |  |  |  |  |  |  |  |  |  |  |
| *5. I.* cf. *lupus* Conchos | 0.005± 0.002 | 0.004± 0.002 | 0.003± 0.002 | 0.003± 0.002 |  |  |  |  |  |  |  |  |  |  |  |
| *6. I.* cf. *lupus* Cuatro Cienegas | 0.007± 0.002 | 0.006± 0.002 | 0.005± 0.002 | 0.005± 0.002 | 0.002± 0.001 |  |  |  |  |  |  |  |  |  |  |
| *7. I. mexicanus* | 0.008± 0.002 | 0.007± 0.002 | 0.007± 0.002 | 0.007± 0.002 | 0.005± 0.002 | 0.007± 0.002 |  |  |  |  |  |  |  |  |  |
| *8. I. australis* | 0.006± 0.002 | 0.005± 0.002 | 0.005± 0.002 | 0.005± 0.002 | 0.003± 0.002 | 0.005± 0.002 | 0.005± 0.001 |  |  |  |  |  |  |  |  |
| *9. I. punctatus* | 0.008± 0.003 | 0.006± 0.003 | 0.006± 0.003 | 0.006± 0.003 | 0.005± 0.002 | 0.007± 0.002 | 0.008± 0.003 | 0.006± 0.002 |  |  |  |  |  |  |  |
| *10. I. balsanus* | 0.017± 0.004 | 0.016± 0.004 | 0.016± 0.004 | 0.016± 0.004 | 0.015± 0.004 | 0.017± 0.004 | 0.018± 0.004 | 0.016± 0.004 | 0.013± 0.004 |  |  |  |  |  |  |
| *11. I. furcatus* | 0.016± 0.004 | 0.015± 0.004 | 0.015± 0.004 | 0.015± 0.004 | 0.014± 0.004 | 0.016± 0.004 | 0.017± 0.004 | 0.015± 0.004 | 0.012± 0.004 | 0.004± 0.002 |  |  |  |  |  |
| *12. I. meridionalis* | 0.017± 0.004 | 0.016± 0.004 | 0.015± 0.004 | 0.016± 0.004 | 0.014± 0.004 | 0.016± 0.004 | 0.018± 0.004 | 0.016± 0.004 | 0.013± 0.004 | 0.005± 0.002 | 0.002± 0.001 |  |  |  |  |
| *13. I.* cf*. lupus* Soto La Marina | 0.005± 0.002 | 0.004± 0.002 | 0.004± 0.002 | 0.004± 0.002 | 0.001± 0.0003 | 0.003± 0.001 | 0.006± 0.002 | 0.004± 0.002 | 0.005± 0.002 | 0.015± 0.004 | 0.014± 0.004 | 0.014± 0.004 |  |  |  |
| *14. Ictalurus* sp. Mezquital | 0.003± 0.001 | 0.001± 0.0005 | 0.0011± 0.0004 | 0.001± 0.000 | 0.004± 0.002 | 0.006± 0.002 | 0.008± 0.002 | 0.006± 0.002 | 0.007± 0.003 | 0.017± 0.004 | 0.016± 0.004 | 0.016± 0.004 | 0.005± 0.002 |  |  |
| *15. I.* cf. *dugesii* Santiago | 0.002± 0.001 | 0.0005± 0.0003 | 0.0002± 0.0002 | 0.0003± 0.0002 | 0.003± 0.002 | 0.005± 0.002 | 0.007± 0.002 | 0.005± 0.002 | 0.006± 0.003 | 0.016± 0.004 | 0.015± 0.004 | 0.015± 0.004 | 0.004± 0.002 | 0.001± 0.000 |  |
| *16. Ictalurus* sp. Nazas | 0.005± 0.002 | 0.004± 0.002 | 0.004± 0.002 | 0.004± 0.002 | 0.004± 0.002 | 0.006± 0.002 | 0.008± 0.002 | 0.006± 0.002 | 0.006± 0.002 | 0.016± 0.004 | 0.015± 0.004 | 0.016± 0.004 | 0.005± 0.002 | 0.005± 0.002 | 0.004± 0.002 |

**Figure S1** Maximum likelihood hypothesis of the genus *Ictalurus* based on the mitochondrial gene *cox1*. mt/nuc discordance in *I. australis* x *I. punctatus* (*a x p*) and *I. lupus* x *I. punctatus* (*l x p*), as well as non-native populations (*^I^*) are indicated in tip labels.


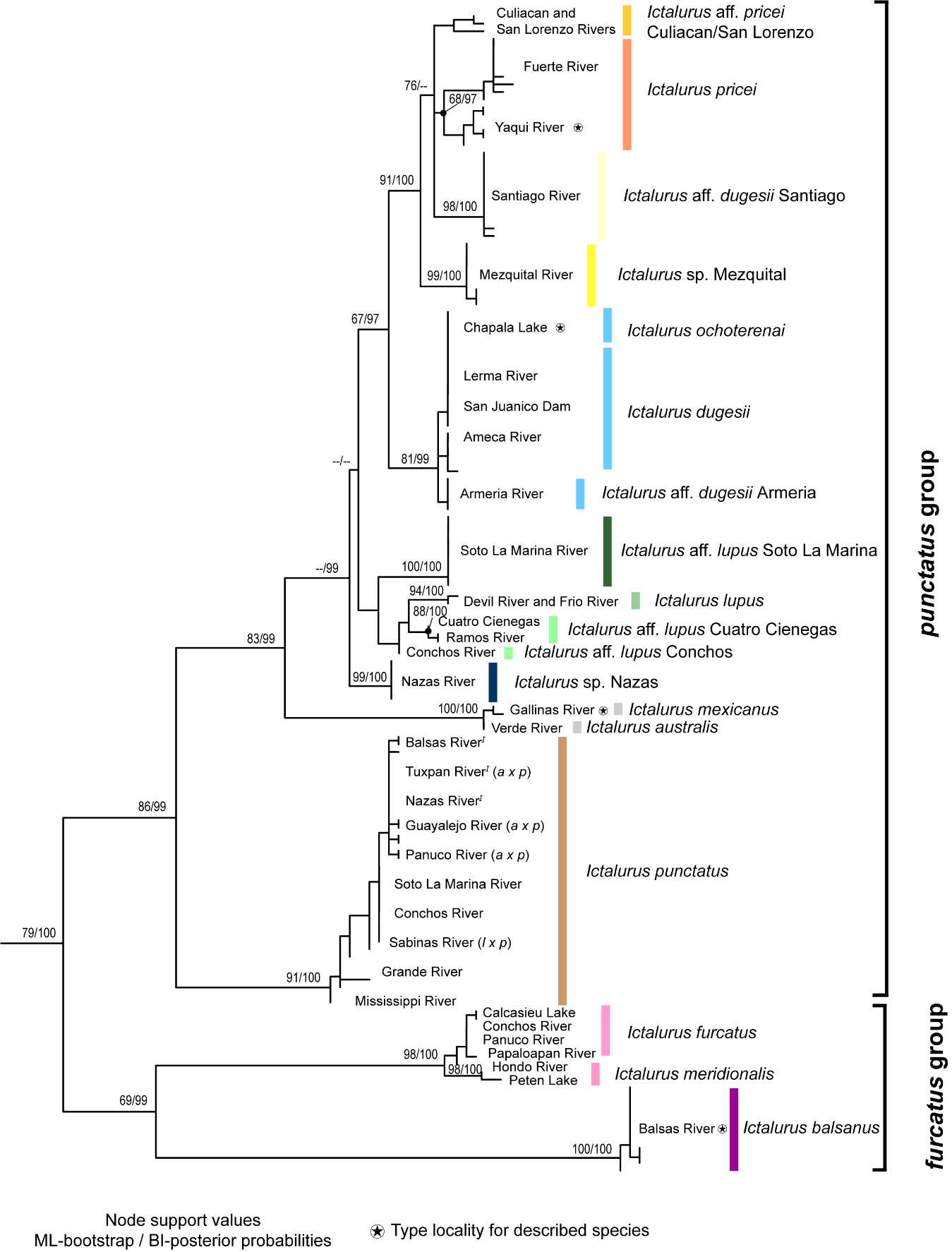


**Figure S2** Maximum likelihood hypothesis of the genus *Ictalurus* based on the nuclear gene *RAG1.* Presumed hybrid *I. australis* x *I. punctatus* (*a x p*), as well as non-native populations (*^I^*) are indicated in tip labels.

**
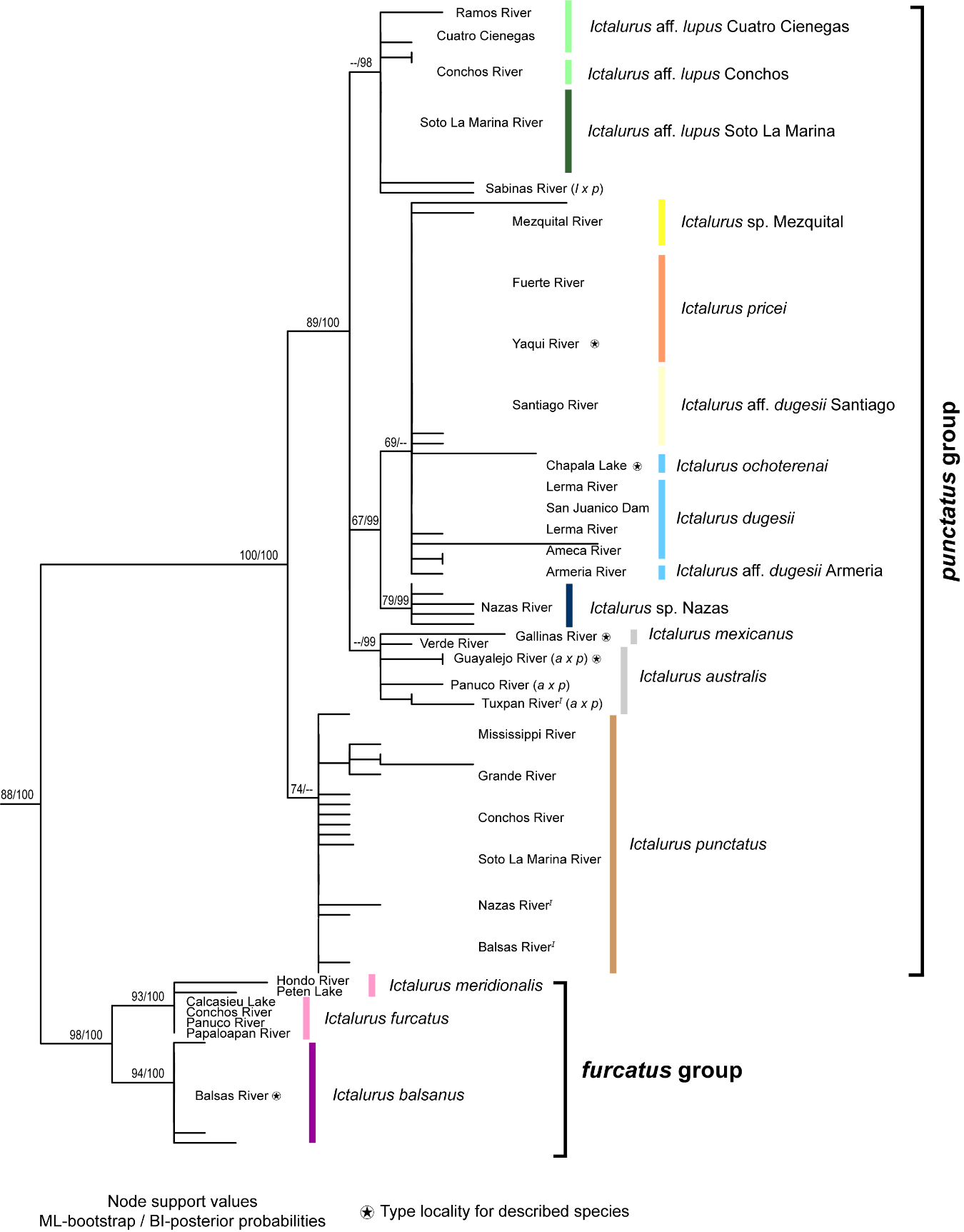
**

**Figure S3.** Maximum likelihood hypothesis of the genus *Ictalurus* based on the mitochondrial gene *atpase8/6.* mt/nuc discordance *I. australis* x *I. punctatus* (*a x p*) and *I. lupus* x *I. punctatus* (*l x p*), as well as non-native populations (*^I^*) are indicated in tip labels.

**
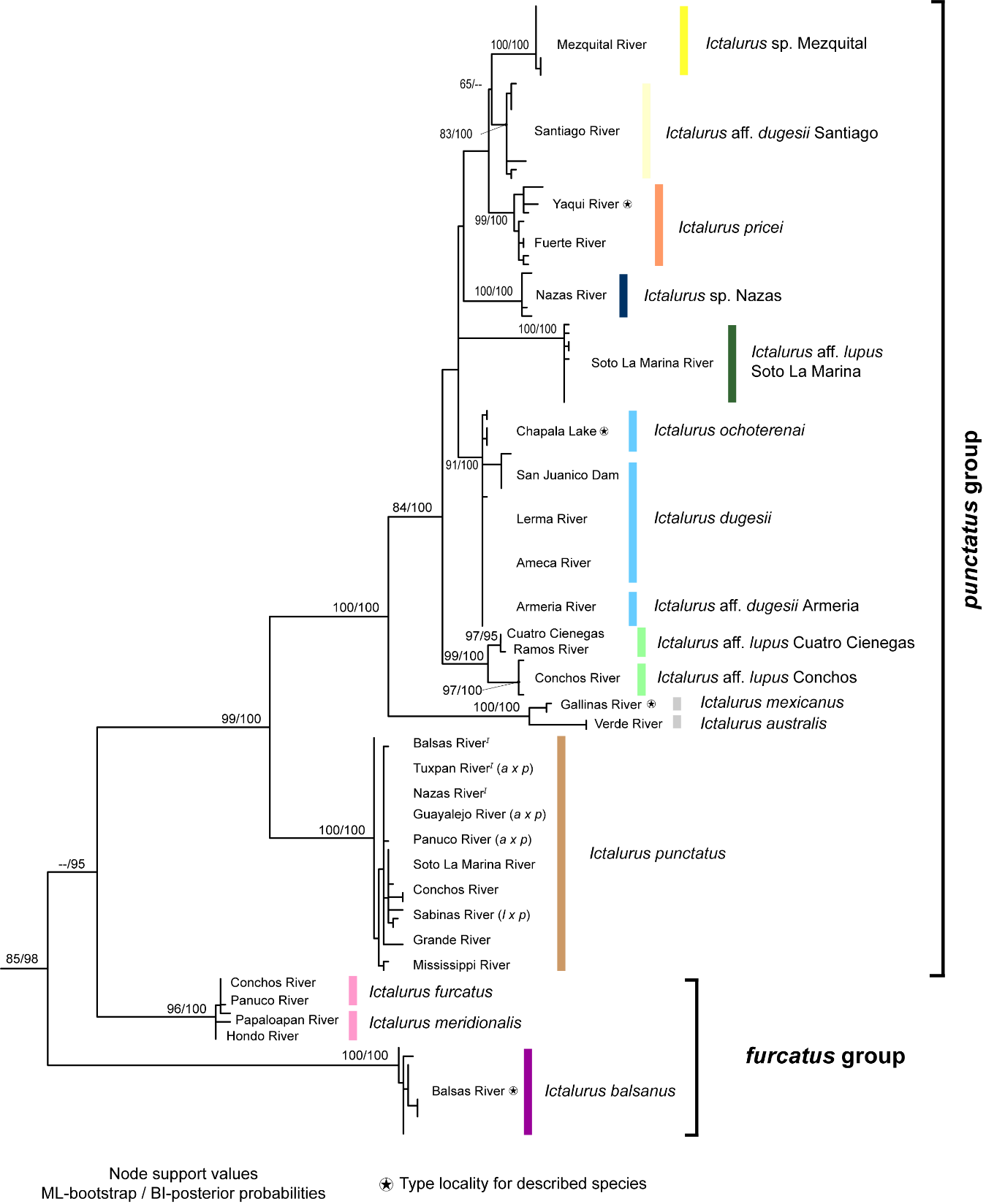
**

**Figure S4.** Time calibrated tree of the genus *Ictalurus*, using BEAST. Node values indicate the age in millions of years; Blue node bars correspond to the 95% highest posterior density.


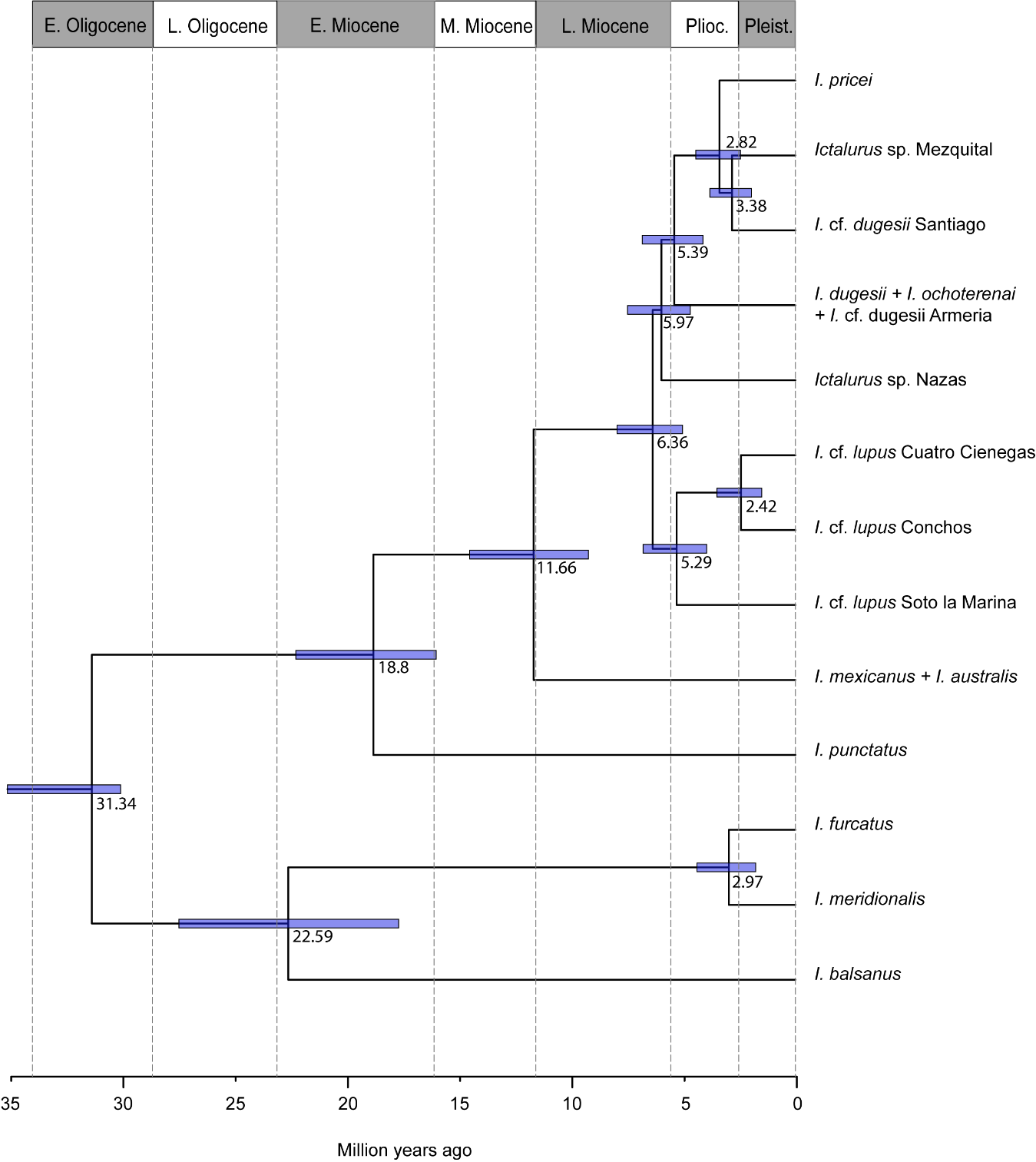

Supplement: Supplementary file 1 — Supplementary Material 1 [file 12862_2023_2134_MOESM1_ESM.docx]
